# Understanding and addressing resistance to IMiDs immunomodulatory compounds in multiple myeloma

**DOI:** 10.1111/febs.70599

**Published:** 2026-05-21

**Authors:** Maria‐Cynthia Fuentes‐Lacouture, Devi Nandana, Karthik Ramasamy, Anjan Thakurta

**Affiliations:** ^1^ Hospital Militar Central De Bogotá Bogotá Colombia; ^2^ Oxford Translational Myeloma Centre, Botnar Research Centre, University of Oxford Oxford UK

**Keywords:** CELMoDs, combination therapies, epigenetic agents, immunomodulatory drugs (IMiDs), multiple myeloma, therapy resistance

## Abstract

Immunomodulatory drugs (IMiDs), including lenalidomide and pomalidomide in combination with proteasome inhibitors, dexamethasone and anti‐CD38 monoclonal antibodies, play a central role in the treatment of multiple myeloma (MM) across newly diagnosed and relapsed stages. These treatment regimens have significantly improved patient outcomes worldwide, establishing IMiDs as one of the backbones of MM therapy. A new generation of more potent compounds called cereblon E3 ligase modulators (CELMoDs) is now being developed to potentially replace the older IMiDs. In addition, novel immunotherapeutic approaches led by chimeric antigen receptor (CAR T), T‐cell engagers and antibody–drug conjugates are also increasingly used in relapsed and refractory myeloma patient care. However, despite these advances, resistance to IMiD‐based therapies inevitably develops and represents a major clinical challenge. Understanding the biological basis of resistance to IMiD‐based therapy is crucial to plan and maximise treatment options for patients when they relapse on IMiD containing regimens. Emerging evidence underscores the role of genetic and epigenetic alterations, changes in downstream signalling, and dysregulation of the bone marrow immune microenvironment in driving therapeutic resistance. In this review, we explore current literature on the molecular and immune mechanisms related to the onset of therapeutic resistance. We then suggest ways to overcome resistance and exemplify options for the future, focusing on immunotherapy combinations with IMiDs or CELMoDs and novel agents.

## Graphical abstract

IMiDs are pivotal in the treatment of multiple myeloma. Mechanisms of resistance comprise cell intrinsic and extrinsic pathways, involving tumour microenvironment, immune cell dysfunction, CRBN‐dependent and independent genetic drivers and epigenetic changes. Recognising these mechanisms of resistance permits the identification of new treatment modalities, as well as facilitates the understanding of pathways for new drug development to treat relapsed and refractory multiple myeloma patients.

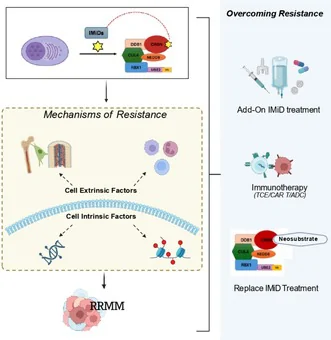

**Abbreviations**
ADC: antibody–drug conjugate
ADO: adenosine
APOBEC: apolipoprotein B mRNA editing enzyme catalytic polypeptide‐like
ATG: autophagy‐related protein
BIRC5: baculoviral inhibitor of apoptosis repeat‐containing 5
BM: bone marrow
BMSC: bone marrow stromal cell
BPd: belantamab mafodotin‐pomalidomide‐dexamethasone
BRD4: bromodomain‐containing protein 4
Bregs: regulatory B cells
CAM‐DR: cell adhesion‐mediated drug resistance
CAR T: chimeric antigen receptor T cell
CCL5: C‐C motif chemokine ligand 5
CCR7: C‐C chemokine receptor type 7
CDK6: cyclin‐dependent kinase 6
CELMoD: cereblon E3 ligase modulating drug
CoMMpass: Multiple Myeloma Research Foundation study database
COP9: constitutive photomorphogenesis 9 signalosome
CRBN: cereblon
CRL4CRBN: CUL4‐RBX1‐DDB1‐CRBN E3 ubiquitin ligase complex
CUL4: Cullin‐4
DC: dendritic cell
DEX/d: dexamethasone
DNAM‐1: DNAX accessory molecule‐1
DRd: daratumumab‐lenalidomide‐dexamethasone
DUOX2: dual oxidase 2
DVRd: daratumumab‐bortezomib‐lenalidomide‐dexamethasone
ECM: extracellular matrix
ETV4: E‐twenty‐six variant transcription factor 4
EZH2: enhancer of zeste homologue 2
FAM72: family with sequence similarity 72
FcRH5: Fc receptor‐like 5
FGF: fibroblast growth factor
FOXP3: Forkhead Box P3
GM‐CSF: granulocyte–macrophage colony‐stimulating factor
GPRC5D: G protein‐coupled receptor class C group 5 member D
H3K27Ac: histone H3 lysine 27 acetylation
H3K27me3: histone H3 lysine 27 trimethylation
H3K36me2: histone H3 lysine 36 dimethylation
HIF1α: hypoxia‐inducible factor 1‐alpha
HLA: human leukocyte antigen
ICAM‐1: intercellular adhesion molecule 1
ICOS: inducible T‐cell costimulator
ID: immune depleted
IDH1: isocitrate dehydrogenase 1
IE: immune excluded
IFN‐γ: interferon‐gamma
IGF1: insulin growth factor 1
IKZF: Ikaros family zinc finger proteins
IL: interleukin
IMiD: immunomodulatory drug
IP: immune permissive
IR: immune resistant
IRF4: interferon regulatory factor 4
IS: immune suppressed
Isa: isatuximab
Isa‐KRd: isatuximab‐carfilzomib‐dexamethasone
JAK/STAT: Janus kinase/signal transducer and activator of transcription
K: carfilzomib
KLRG1: killer cell lectin‐like receptor G1
LAG‐3: lymphocyte activation gene‐3
LC3: microtubule‐associated proteins 1A/1B light chain 3
Len/R: lenalidomide
LOH: loss of heterozygosity
MCP‐1/2: monocyte chemoattractant protein‐1/2
MDSC: myeloid‐derived suppressor cell
MEK/ERK: Mitogen‐Activated Protein Kinase Kinase/Extracellular Signal‐Regulated Kinase
Mezi: mezigdomide
MGUS: monoclonal gammopathy of undetermined significance
MICA: MHC class I polypeptide‐related sequence A
MIP‐1α: macrophage inflammatory protein‐1 alpha
MM: multiple myeloma
MMP: matrix metalloproteinase
MRD: minimal residual disease
mRNA: messenger ribonucleic acid
MRP: multidrug resistance protein
NDMM: in newly diagnosed multiple myeloma
NEDD8: neural precursor cell‐expressed developmentally downregulated protein 8
NK: natural killer cell
NKT: natural killer T cell
NOS2: nitric oxide synthase 2
NSD2/MMSET: nuclear receptor binding SET domain protein 2
OS: overall survival
PBX1: PD‐1 cell, programmed death‐1 cell, pre‐B‐cell leukaemia transcription factor 1
PFS: progression‐free survival
P‐Gp: P‐glycoprotein
Pom/P: pomalidomide
PTGS2: prostaglandin‐endoperoxide synthase 2
PVd: pomalidomide‐bortezomib‐dexamethasone
RBX1: RING‐box protein 1
RRMM: relapsed/refractory multiple myeloma
RUNX: Runt‐related transcription factor
RVd: lenalidomide‐bortezomib‐dexamethasone
SASP: senescence‐associated secretory phenotype
Sel/S: selinexor
shRNA: short hairpin ribonucleic acid
SMAD3: mothers against decapentaplegic homologue 3
STAT3: signal transducer and activator of transcription 3
TCE: T‐cell engager
Tcm: central memory T cell
TCR: T‐cell receptor
TGF‐β: transforming growth factor beta
TH17: T helper 17 cells
TIGIT: T‐cell immunoreceptor with Ig and ITIM domains
TIM‐3: T‐cell immunoglobulin and mucin domain‐containing protein 3
TME: tumour microenvironment
Tregs: regulatory T cells
UBE2: ubiquitin‐conjugating enzyme E2
V/Vd: bortezomib (alone or in combination with dexamethasone)
VCAM‐1: vascular cell adhesion molecule 1
VEGF: vascular endothelial growth factor
VGPR: very good partial response
WGS: whole genome sequencing
XPO1: exportin‐1

## Introduction

Immunomodulatory drug (IMiD) lenalidomide plays a pivotal role in newly diagnosed multiple myeloma (NDMM) as part of combinational triplet and quadruplet therapeutic regimens. Lenalidomide is also approved for use as monotherapy in the maintenance setting following autologous stem cell transplantation. Pomalidomide, a next‐generation IMiD, is similarly a cornerstone of triplet therapies in relapsed and refractory multiple myeloma (RRMM). Despite their widespread use and clinical effectiveness, most patients eventually relapse and develop resistance to IMiDs and IMiD‐based regimens. The underlying mechanisms driving clinical resistance to IMiDs remain incompletely understood, but growing evidence highlights the role of genetic and immune factors as key contributors. A next generation of more potent IMiD compounds such as Iberdomide and Mezigdomide (also known as CELMoDs) that can partially overcome resistance to older IMiDs is being developed.

**Fig. 1:**
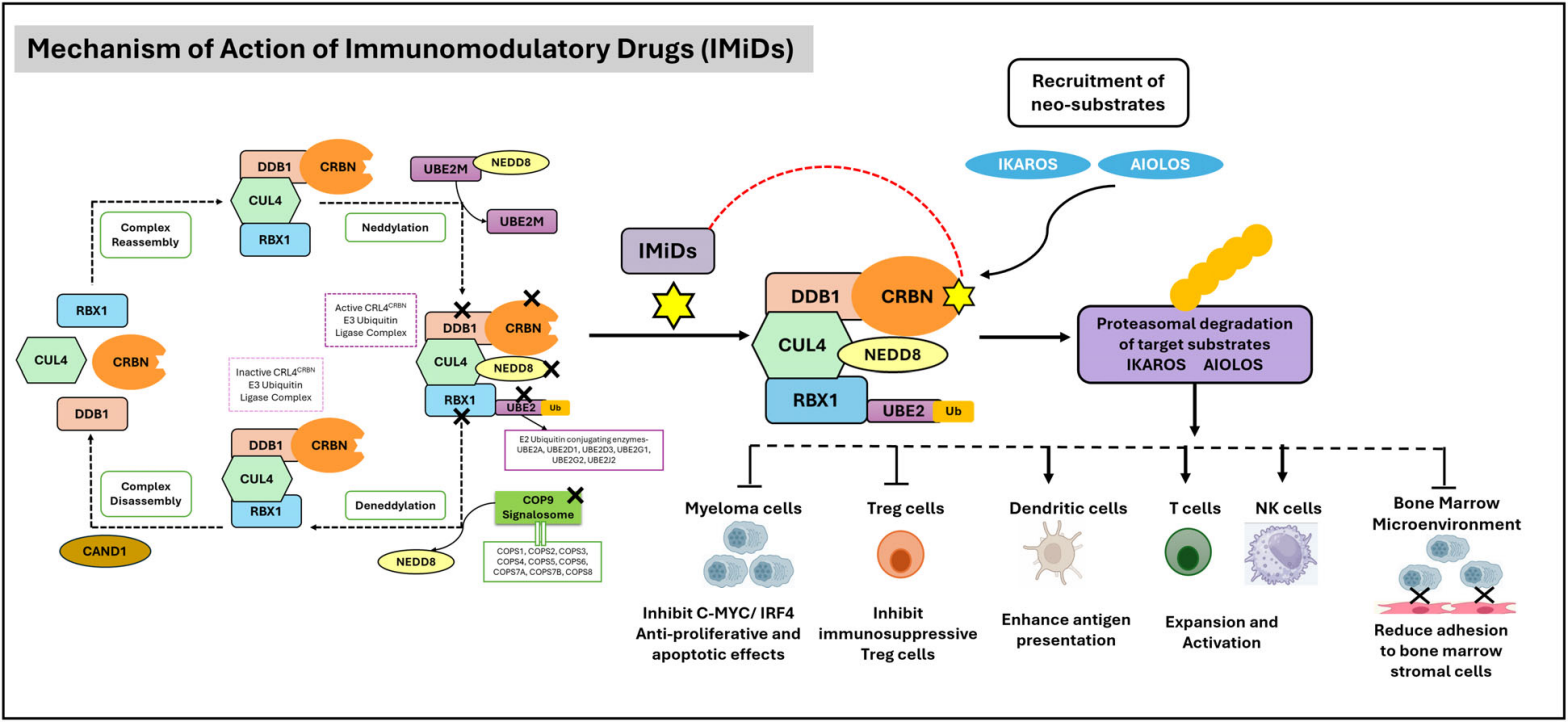
Mechanism of Action of Immunomodulatory Drugs (IMiDs) and mutations related to resistance. The IMiDs function by binding to the cereblon (CRBN), a substrate receptor of the CRL4^CRBN^ E3 ubiquitin ligase complex, comprising CUL4, RBX1, DDB1 and CRBN. The activity of this complex is tightly regulated by the process of neddylation–deneddylation mediated by COP9 signalosome. IMiD binding to the CRBN leads to recruitment and proteasomal degradation of neo‐substrates such as IKAROS and AIOLOS leading to downregulation of their downstream targets such as C‐MYC and IRF4, and thereby exerting antiproliferative effects on MM cells. The immunomodulatory effects of IMiDs include inhibition of the immunosuppressive Tregs, enhanced antigen presentation by dendritic cells, expansion and activation of T and NK cells. IMiDs also reduce adhesion of MM to stromal cells. Aberrations (crossed) affecting CRBN, the other components of the CRL4^CRBN^ E3 ubiquitin ligase complex and the COP9 signalosome have all been linked to IMiD resistance. COP9, Constitutive Photomorphogenesis 9 signalosome; CRBN, Cereblon; CUL4, Cullin‐4; DDB1, Damage‐specific DNA binding protein 1; IRF4, Interferon regulatory factor 4; RBX1, the RING‐finger protein RING‐box1; MM, Multiple Myeloma; NEDD8, Neural precursor cell‐expressed developmentally downregulated protein 8; NK cells, Natural Killer cells; Tregs, regulatory T cells; UBE2, Ubiquitin‐conjugating enzyme E2.

All clinical IMiDs and CELMoD compounds act via a common target Cereblon (CRBN), through engagement with the CUL4‐RBX1‐DDB1‐CRBN (CRL4^CRBN^) E3 ubiquitin ligase complex, comprising the scaffold Cullin‐4 (CUL4), the RING‐finger protein RING‐box1 (RBX1), the adapter protein damage‐specific DNA binding protein 1 (DDB1), and the substrate receptor CRBN. The active/inactive state of the CRL4^CRBN^ E3 ligase is tightly regulated by the process of neddylation/deneddylation mediated by the COP9 signalosome (Fig. 1). Binding of IMiDs to the tri‐tryptophan pocket of CRBN functions like a molecular glue to recruit critical neo‐substrates, most notably the transcription factors Ikaros and Aiolos, targeting them for proteasomal degradation. This rapid degradation cascade results in the suppression of downstream targets such as c‐Myc and IRF4, ultimately leading to antiproliferative effects and apoptosis [1, 2, 3]. In the immune cells, the molecular mechanism leads to immunomodulatory effects on various immune subsets promoting T‐cell expansion and NK cell activation through increased production of IL‐2 and IFN‐γ, while also suppressing the activity of immunosuppressive regulatory T cells (Tregs). Additionally, they enhance antigen presentation by dendritic cells, improving the immune system's ability to recognise and target myeloma cells [4]. Within the bone marrow, IMiDs disrupt the protective interactions between myeloma cells and stromal cells by downregulating adhesion molecules like VCAM‐1 and ICAM‐1. They also impair tumour‐supportive signals by reducing the activity of angiogenic factors such as VEGF and FGF, further weakening the tumour microenvironment [4].

A number of recent reviews describe the landscape of IMiD resistance and how to address them [3, 4, 5]. A previous review authored by one of us described the mechanistic features of IMiDs (lenalidomide and pomalidomide) and CELMoDs (iberdomide and mezigdomide) and suggested ways to differentiate the latter from each other and the IMiDs [6]. Since then, both iberdomide and mezigdomide have advanced in the clinic and studies are underway to demonstrate the efficacy of these drugs in NDMM as well as in RRMM. At the intervening time, a number of new therapeutic agents have been approved in myeloma care, including anti‐BCMA CAR T, T‐cell engagers (TCE), antibody–drug conjugate (ADC), anti‐GPRC5D and anti‐FcRH5 TCE, and XPO1 small molecule inhibitor. Many of these agents are competing for their use in RRMM and potentially in NDMM treatment paradigms. Under this evolving landscape of myeloma care, it is critical to understand the key issues related to IMiD resistance and how to address them therapeutically. And more specifically, what role CELMoD based regimens could play in overcoming IMiD resistance. In this review, we look at the clinical and preclinical evidence around the diversity of contributors, focusing on genetic, epigenetic, immune, and patient‐derived factors and potential mechanisms underlying resistance to this class of drugs. We address the unmet medical need in the context of new and evolving therapeutic landscape and indicate ways to overcome IMiD resistance, especially focusing on the combination of approved drugs with emerging agents in the clinic.

### Cell intrinsic genetic factors

The genetic landscape of IMiD resistance can be broadly categorised into cereblon‐dependent and cereblon‐independent resistance mechanisms.

#### CRBN dependent genetic determinants

On drug exposure, the expression level of *CRBN* mRNA and protein levels generally decrease over time in malignant plasma cells, and patients acquiring lenalidomide resistance show a markedly reduced expression of CRBN [7, 8].

As such, the four cysteine residues in the IMiD binding domain of the CRBN are highly conserved among vertebrates showing their functional importance. The mutations in these cysteine residues can critically impact the zinc finger domain formation resulting in protein misfolding and aggregation [9, 10]. Interestingly, there was a significant increase in exon 10 spliced variant of *CRBN* in pomalidomide RRMM patients compared to NDMM patients [11]. The exon‐10 splice variant of CRBN lacks the critical tri‐tryptophan binding pocket encoded by exons 10–11, hence impairing the IMiD interaction with the ligase complex. Functional validation in myeloma cell lines confirmed that exon 10 exclusion alone was sufficient to abrogate lenalidomide‐induced cytotoxicity, highlighting its direct role in mediating resistance [12]. A recent study has also pointed out the dual deletion of exon 8 and 10 in NDMM and IMiD‐refractory patients, although the exact role of this variant in IMiD resistance is yet to be deciphered [13].

In a study of 50 RRMM patients who were pretreated with IMiDs, there was a 12% and 10% increased prevalence of mutations in *CRBN* and CRBN pathway genes, respectively, when compared to NDMM [14]. A more recent study using whole genome sequencing (WGS) data from 455 patients and RNAseq data from 655 patients, IMiD‐refractory patients exhibit a significantly higher incidence of *CRBN*‐related genetic aberrations including mutations in the *CRBN* gene, gene copy loss of *CRBN* and the presence of exon 10‐spliced variant of *CRBN*, when compared to NDMM [11]. Additionally, the cumulative proportion of *CRBN* alterations increased from lenalidomide‐resistant patients (20%) to pomalidomide‐resistant patients (30%). Importantly, the genetic aberrations of *CRBN* significantly correlated with a reduced overall survival and progression‐free survival (PFS) [11]. In the Iberdomide phase 1 study, Amatangelo *et al*. reported that among 81 late line RRMM patients, *CRBN* exon 10 splicing was detected in 15%, loss of heterozygosity (LOH) at the *CRBN* locus in 14%, and *CRBN* mutations in 6%, highlighting the prevalence of *CRBN* alterations in RRMM. Collectively, *CRBN* disruption is one of the most prominent genetic contributors to clinical resistance in RRMM upon acquiring IMiD resistance [15].

#### CRBN independent genetic determinants

Initial analysis of some pomalidomide‐resistant cell lines had no loss in CRBN expression [1], indicating a CRBN‐independent pathway for IMiD resistance. Emerging evidence from genomics analysis also suggests that resistance to IMiDs can also occur through CRBN‐independent mechanisms in patients.

COP9 Signalosome plays an important role in the neddylation/deneddylation of CRL4^CRBN^ E3 ubiquitin ligase complex. The conjugation of NEDD8 stimulates the CRL4^CRBN^ complex into an active state whereas the COP9 signalosome mediated removal of NEDD8 switches the complex back into an inactive state [16]. A specific chromosomal region, 2q37, harbouring COP9 signalosome components *COPS7B* and *COPS8*, was found to exhibit increasing copy loss frequency across disease stages, from 5.5% in newly diagnosed patients to 10.0% in those refractory to lenalidomide and rising to 16.4% in patient's refractory to both lenalidomide and pomalidomide [17]. In an independent cohort of 50 patients with serial samples collected during treatment, 2q37 deletion emerged in 16% of cases exposed to IMiDs, while it was absent in patients without IMiD treatment [17]. In the Iberdomide phase I study, WGS and RNAseq data from 81 patients revealed that 17% had loss of *COPS7B* and 16% had loss of *COPS8*, further underscoring the therapeutic significance of the 2q37 chromosomal region, where both genes are located [15].

As such, the intrinsic fitness of the myeloma clone can significantly contribute to drug resistance. Analysis of the myeloma genomes of 386 RRMM patients resistant to the IMiDs also revealed the enrichment of mutations in *DUOX2, EZH2, TP53* and gain in 1q, del17p and double hit events that are associated with high risk, suggesting the contribution of other genomic or epigenomic factors could drive resistance to the IMiDs [18].

Indeed, pathway analysis in resistant myeloma cell lines revealed enrichment of the JAK/STAT signalling pathway, with *IL6* and *STAT3* among the most upregulated genes. Increased expression of STAT3 and its downstream targets PIM2 and BIRC5 was confirmed in XG1 lenalidomide‐resistant cells. Moreover, IL6 exposure induced lenalidomide resistance in wild‐type XG1 cells, supporting the role of IL6‐driven STAT3 activation in mediating resistance [19].

Elevated c‐MYC protein levels have been observed at lenalidomide‐refractory stages, compared to diagnosis, implying a potential role of c‐MYC in driving resistance [7]. Among other preclinical evidence, truncated IRF4 is resistant to lenalidomide‐induced downregulation [19].

Furthermore, in an *in vivo* model of acquired resistance, Ocio *et al*. found that resistance to lenalidomide–dexamethasone and pomalidomide–dexamethasone was associated with activation of the MEK/ERK pathway [20]. Also, overexpression of CDK6 in multiple myeloma (MM) cell lines resulted in markedly reduced IMiD sensitivity. Inhibiting CDK6 demonstrates strong synergy with IMiDs both *in vitro* and *in vivo* [21]. Notably, this resistance mechanism is kinase‐dependent, as expression of a catalytically inactive *CDK6* mutant (K43M) failed to protect cells from lenalidomide‐induced cytotoxicity [21]. Zhou *et al*. identified RUNX1 and RUNX3 as transcription factors that bind to Ikaros and Aiolos, protecting them from CRBN‐mediated degradation and thereby reducing IMiD efficacy [22].

Finally, gene expression profiling and pathway analysis on lenalidomide‐resistant cell lines developed through chronic exposure to IMiDs showed dysregulation of Wnt/β‐catenin pathway [23]. Activation of the Wnt/β‐catenin pathway either through recombinant Wnt‐3a or β‐catenin overexpression diminished the antiproliferative effects of lenalidomide. In contrast, silencing β‐catenin using shRNAs re‐established drug sensitivity in plasma cells [23]. Lenalidomide‐resistant myeloma models exhibited elevated expression of CD44, a Wnt/β‐catenin target, correlating with increased adhesion to bone marrow stroma and hyaluronan‐coated surfaces, showing how adhesion pathways drive tumour microenvironment (TME)‐mediated IMiD resistance [24].

These observations taken together confirm drug mechanism‐based, CRBN‐dependent and CRBN‐independent genetic drivers of resistance in myeloma in cell lines and IMiD‐resistant patients.

### Immunological mechanisms driving IMiD resistance

**Fig. 2:**
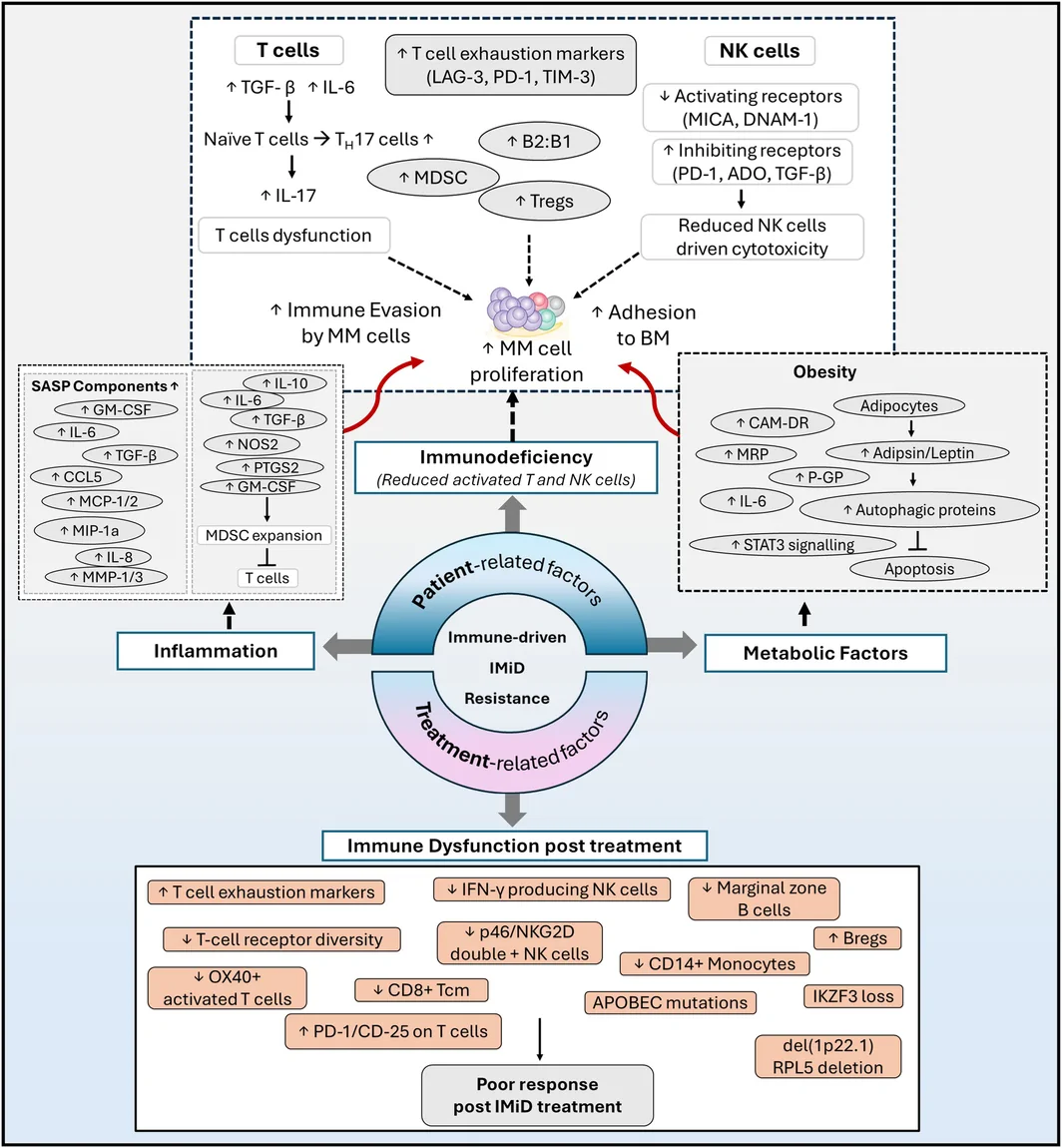
Immune‐mediated IMiD resistance. Schematic overview of patient‐related and treatment‐related factors contributing to immune dysfunction and IMiD resistance. Patient‐related contributors include inflammation, metabolic syndrome/obesity and the state of immunodeficiency at diagnosis which leads to immune evasion strategies by MM cells leading to reduced T and NK cell activity. Treatment‐related factors include immune dysfunction post‐treatment characterised by T‐cell exhaustion, impaired NK cell cytotoxicity, altered monocyte and B‐cell subsets, and genetic alterations such as IKZF3 loss, APOBEC mutations and del(1p22.1) affecting RPL5. Collectively, these factors drive poor responses to IMiD therapy and highlight opportunities for novel immune‐restorative strategies. ADO, adenosine; APOBEC, apolipoprotein B mRNA editing enzyme catalytic polypeptide‐like; BM, bone marrow; Bregs, B regulatory cells; CAM‐DR, cell adhesion‐mediated drug resistance; CCR7, C‐C chemokine receptor type 7; CCL5, C‐C motif chemokine ligand 5; del(1p22.1), deletion of chromosome 1p22.1; DNAM1, DNAX accessory molecule‐1; GM‐CSF, granulocyte–macrophage colony‐stimulating factor; IFN‐γ, interferon‐gamma; IKZF3, IKAROS family zinc finger protein 3; IL, interleukin; IMiD, immunomodulatory drug; LAG‐3, lymphocyte activation gene‐3; MCP‐1/2, monocyte chemoattractant protein‐1/2; MDSC, myeloid‐derived suppressor cell; MICA, MHC class I polypeptide‐related sequence A; MIP‐1α, macrophage inflammatory protein‐1 alpha; MMP, matrix metalloproteinase; MRP, multidrug resistance protein; NK, natural killer; NKG2D, Natural Killer group 2, member D; NKT, natural killer T cell; NOS2, nitric oxide synthase 2; PD‐1, programmed cell death protein 1; P‐GP, P‐glycoprotein; PTGS2, Prostaglandin‐endoperoxide synthase 2; SASP, senescence‐associated secretory phenotype; STAT3, signal transducer and activator of transcription 3; TGF‐β, transforming growth factor beta; T_H_ cells, T helper cells; TIGIT, T‐cell immunoreceptor with Ig and ITIM domains; TIM‐3, T‐cell immunoglobulin and mucin domain‐containing protein 3; Treg, regulatory T cell.

Immunological mechanisms causing IMiD resistance include patient‐related factors—where the systemic characteristics (inflammatory, metabolic and immunological) of the patients at diagnosis can modulate response to IMiDs, and treatment‐related factors— where the changes in the immune system after exposure to IMiDs drive the resistance (Fig. 2).

#### Patient‐related factors: Baseline immune characteristics

##### State of immunodeficiency at diagnosis

The alterations of the immune profile in patients with MM, which may begin early in myelomagenesis, have allowed the description of immunophenotypes in patients with the disease, based mainly on the distribution of dendritic cells (DCs) and T cells and its correlation with patients' outcomes and responses to treatment. This classifies the patients into 5 subgroups: immune depleted (ID), immune permissive (IP), immune excluded (IE), immune suppressed (IS) and immune resistant (IR) [25]. Dysregulation of the immune system due to an imbalance in the regulatory and cytotoxic equilibrium is one of the predominant biological mechanisms in MM, contributing to the chronic course of the disease and its frequent relapses [26, 27]. In NDMM patients, baseline immunological characteristics that have been demonstrated to contribute to this myelomagenesis include an increase in CD4+ and CD8+ T cells [28, 29, 30], and in the T helper T_H_17 cells, which is a subset of CD4+ T cells that have an immunosuppressive phenotype. This is driven by increased levels of cytokines such as TGF‐β and IL‐6 in the bone marrow microenvironment which induces the differentiation of naïve T cells to T_H_17 cells. These T_H_17 cells induce production of IL‐17 which further promotes MM cell proliferation, colony formation and adhesion to bone marrow stromal cells (BMSCs) (Fig. 2). Supporting all these, both T_H_17 cell population as well as IL‐17 levels were elevated in the peripheral blood and bone marrow of NDMM patients [31] Similarly, there is an enrichment of NK cells in NDMM patients compared to healthy individuals [28, 30, 32].

At first relapse, there is an increase in the proportion of plasmacytoid DCs, T_H_17 cells and myeloid‐derived suppressor cells (MDSC), along with dysfunction of NK cells, and an elevated ratio of B2 to B1 cells [27]. Particularly, CD11b^+^CD14^−^HLA^−^DR^−/low^CD33^+^CD15^+^ MDSCs are significantly increased in both the peripheral blood and the bone marrow of patients compared with healthy donors, which stimulate MM growth while suppressing T‐cell‐mediated immune responses [33].

However, as the disease progresses, the cytotoxic function of NK cells declines due to reduced expression of activating receptors such as MICA, DNAM‐1, increased PD‐1 expression and the influence of immunosuppressive cytokines like ADO and TGF‐β. These changes contribute to immune evasion by myeloma cells [28]. These last changes adopt a predictive value as lenalidomide‐refractory patients show a significant reduction in the proportion of activated NK cells and effector T cells [34]. Other changes predictive of response with IMiD include immune cell levels, as described in the analysis of baseline immune characteristics of a subset of RRMM patients (len/pom or triple‐class refractory) from the phase 1/2 iberdomide study; median absolute counts of several immune cell types including the B cells, total T cells and CD4+ T cells were below the lower limit of normal [15]. Also, despite an ‘activated immunophenotype’, a low proportion of T cells were observed to be in a proliferative state, suggesting immune dysfunction. These findings were accompanied by an increase in T cells (Tregs) and T‐cell exhaustion markers, specifically Lag‐3 (CD223), PD‐1 (CD279) and Tim‐3 (CD366) [15]. Therefore, the baseline immune characteristics including dysfunctional NK cells and immunosuppressive T‐cell profiles could manifest as resistance or reduce the impact of IMiD‐based therapy in MM.

##### Role of inflammation

An intact immune microenvironment is essential for controlling myeloma. Given that most MM patients are older adults, both inflammaging (age‐related proinflammatory changes in the bone marrow microenvironment) and immune cell senescence play important roles in tumour development and growth mechanisms [35, 36].

In the TME of MM patients, several *senescence‐associated secretory phenotype* (SASP) components have been found to be elevated, particularly IL‐6, IL‐8, TGF‐ β, CCL5, MIP‐1a, MMP‐1/3, MCP‐1/2, among others [37]. These SASPs, produced in an autocrine manner by MM cells, have a direct trophic effect on neoplastic cells, promoting their proliferation.

Additionally, elevated IL‐6, GM‐CSF and factors such as PTGS2, TGFβ, NOS2 and IL‐10 from mesenchymal cells promote MDSC expansion, which suppresses T‐cell function by producing reactive oxygen species, arginase and nitric oxide synthase [38, 39, 40]. These mechanisms of immunosenescence are relevant as IMiDs downregulate the MDSC‐promoting chemokines such as CCL5, MIF, to control the disease [41]. However, some studies report increased MDSC‐like CD14^+^CD15^+^ cells after treatment with lenalidomide, which suppress T‐cell activity and may contribute to drug resistance [42].

##### Metabolic factors

Metabolic syndrome, particularly obesity, is associated with increased incidence and worse outcomes for MM patients. The risk is higher in individuals categorised as overweight or obese compared to those of normal weight, and obesity also increases the risk of progression from MGUS to MM [43, 44]. In the same metanalysis, relative risk estimates for mortality were also higher for obese patients compared to patients with normal body mass index. Similarly, Ochiai, *et al*. demonstrated that adipocytes from overweight and obese individuals increased cell adhesion‐mediated drug resistance (CAM‐DR), P‐glycoprotein (P‐gp) and multidrug resistance‐associated protein (MRP) transporter expression in MM cells [45]. Furthermore, myeloma cell lines cocultured with adipocytes proliferated faster and displayed increased pSTAT‐3/STAT‐3 signalling in adipocyte‐conditioned media [46]. Interestingly, adipocytes from obese patients had higher production of other inflammatory proteins, including IL‐6 [46]. Among other mechanisms, the contact between tumour cells and adipocytes in bone marrow leads to the secretion of adipokines such as adipsin and leptin (typically seen in obese MM patients), which further upregulate autophagic proteins such as ATG3, ATG5 and LC3‐1/II via JAK/STAT signalling and inhibit caspase cleavage and apoptosis in MM cells [47].

#### Treatment‐related factors: Pharmacodynamic changes after exposure to IMiDs

The disease evolution takes a multistep trajectory whereby the immune system exerts selective pressure on tumour cells through a process known as immunoediting. The first stage, ‘elimination’ involves the innate and adaptive immune system‐mediated killing of transformed cells that have initially escaped the apoptotic/senescence checkpoints. The second stage, ‘equilibrium’ involves the expansion of those resistant tumour subclones that have evaded the elimination stage through various genetic alterations. Even at this stage, the net tumour growth is controlled by the adaptive immunity. Eventually, in the final ‘escape’ stage, the selective pressure on tumour subclones leads to their outgrowth and establishment of a clinically apparent immunosuppressive tumour microenvironment [48, 49].

Emerging evidence highlights that IMiD resistance is closely linked to impairment of immune effector functions during treatment. Although pomalidomide exerts pleiotropic effects on multiple immune cell populations, not all of these changes translate into clinical benefit. Immune profiling of peripheral blood samples collected at screening, cycle 1 day 8, cycle 3 day 8 post‐treatments from the CC4047‐MM‐007 (OPTIMISMM, NCT01734928) study involving 186 patients treated with pomalidomide in combination with bortezomib and dexamethasone (PVd) demonstrated significant enhancements in NK, NKT and T‐cell activation compared to those receiving only Vd. Specifically, increased double positive expression of NK cell activation markers (p46/NKG2D), increased OX‐40+ activated CD8^+^ T cells (OX‐40^+^), decreased PD‐1 and CD25 expression on CD4^+^ T cells, expansion of marginal zone B‐cell subsets, and decrease in B regulatory cells were all associated with significantly better PFS among PVd treated patients [27].

Based on RRMM patients from a phase 2 trial (NCT01319422, n = 39) treated with pom (received prior len therapy), treatment induced increases in total T cells, particularly CD8^+^ T cells, correlated with objective therapeutic responses, whereas NK cell expansion did not. Moreover, clinical response was specifically associated with Pom‐induced elevations in IFNγ‐ and TNFα‐producing CD8^+^ T cells and polyfunctional T‐cell populations, highlighting the importance of robust cytotoxic T‐cell activation. In contrast, treatment induced changes in T cells producing other cytokines (IL‐13 and IL‐17), cytolytic markers (granzyme, perforin), FOXP3^hi^ Tregs, or NK cell numbers did not significantly correlate with response [50].

Pierceall *et al*. analysed whether baseline immune cell profiles and their early changes during treatment were linked to outcomes in MM. In this study, immune cell phenotyping was performed both at baseline and at cycle 2 day 15 (C2D15) to evaluate associations with PFS (relapsed vs. nonrelapsed patients) and depth of response (≥very good partial response [VGPR] vs. <VGPR). At the 18‐month PFS, higher baseline counts of total CD4^+^ T cells and specific CD4^+^ subsets, such as naïve, central memory (Tcm) and activated ICOS^+^ cells, were significantly associated with longer PFS and better response rates (≥VGPR). Elevated CD4^+^ counts after treatment start also correlated with improved outcomes. While CD8^+^ T‐cell counts did not significantly relate to PFS, higher baseline and post‐treatment CD8^+^ Tcm counts were significantly associated with deeper responses. NK cells showed a trend toward PFS benefit, but no associations were found for B cells, monocytes, Tregs or exhausted T cells [34].

As such, MM progression during treatment is shaped by a dynamic interaction between the tumour and the immune microenvironment. Analysis of the MM tumour microenvironment (TME) in patients enrolled in the MANHATTAN trial (NCT03290950; *n* = 49) with NDMM treated with carfilzomib–lenalidomide–dexamethasone–daratumumab combination revealed that those who failed to achieve minimal residual disease (MRD) negativity had significantly shorter PFS [51, 52]. These patients had multiple immune dysregulations such as a decrease in CD14^+^ monocytes, increased markers of T‐cell exhaustion, persistence of IFNγ‐producing natural killer (NK) cells, and diminished T‐cell receptor (TCR) diversity. Notably, these immune alterations co‐occurred with genomic abnormalities such as elevated APOBEC mutational signatures, deletions at 1p22.1 (RPL5), and loss of IKZF3 [52].

In heavily pretreated IMiD‐refractory patients, immune dysfunction was evident, characterised by reduced T‐cell activation and proliferation, diminished cytokine secretion and impaired NK cell activity. Subsequent treatment with Iberdomide resulted in immune restimulation noted by an increase in activated NK and T cells. However, consistent with the role of Ikaros and Aiolos in B‐cell maturation, iberdomide treatment led to a decrease in B‐cell population possibly driven by the degradation of these transcription factors [15]. Similarly, treatment with mezigdomide in heavily pretreated RRMM patients was associated with immune cells switching from exhausted to activated phenotype, across both adaptive and innate compartments [53, 54]. NK and NKT cells significantly increased, while CD4^+^ and CD8^+^ T cells shifted toward an effector memory phenotype with increased HLA‐DR and ICOS expression, indicating enhanced activation. Exhausted and senescent populations such as KLRG1^+^ and CD57^+^ subsets, as well as TIGIT‐expressing CD8^+^, NK and NKT cells, were markedly reduced, with a dose‐dependent decline in TIGIT^+^ populations. Although PD‐1 expression alone was unchanged, dual PD‐1/TIGIT–positive T cells decreased significantly. Importantly, patients who failed to respond to mezigdomide had higher baseline frequencies of PD‐1^+^ and TIGIT^+^ CD8^+^ and NKT cells [53].

These deficits, likely arising from the combined effects of chronic IMiD exposure and tumour‐driven immunosuppression, impair effector immune cell responsiveness to subsequent IMiD‐mediated costimulation. Collectively, these findings highlight that both baseline immune status and therapy‐induced immune perturbations critically influence IMiD resistance in multiple myeloma. A summary of immunological changes correlating with response is described in Table 1.

**Table 1:** Immunological changes correlating with response to immunomodulatory drugs (IMiDs).

Changes correlating with response	References
Elevations in IFNγ‐ and TNFα‐producing CD8^+^ T cells and polyfunctional T‐cell populations	[50]
Higher baseline counts of total CD4^+^ T cells and specific CD4^+^ subsets, such as naïve, central memory (Tcm), and activated ICOS^+^ cells Elevated CD4^+^ counts after treatment start Higher baseline and post‐treatment CD8^+^ Tcm counts	[33]
Increased double‐positive expression of NK cell activation markers (p46/NKG2D) Increased OX‐40+ activated CD8^+^ T cells (OX‐40^+^) Decreased PD‐1 and CD25 expression on CD4^+^ T cells Expansion of marginal zone B‐cell subsets Decrease in B regulatory cells	[27]
Higher baseline frequencies of PD‐1^+^ and TIGIT^+^ CD8^+^ and NKT cells	[53]

Changes that may correlate with lack of response	References
Production of IL‐13 and IL‐17, cytolytic markers (granzyme, perforin) FOXP3^hi^ Tregs NK cells expansion alone	[50]
Levels of B cells, monocytes, Tregs, or exhausted T cells	[33]
Decrease in CD14^+^ monocytes Increased markers of T‐cell exhaustion Persistence of IFNγ‐producing natural killer (NK) cells Diminished T‐cell receptor (TCR) diversity	[52]

### Epigenetic drivers of resistance

Epigenetic plasticity serves as an adaptive strategy through which MM cells evade IMiD treatment, with such mutations comprising almost a quarter of the genes mutated in NDMM, including mutations in *EP300, IDH1 and 2, KMT2B* and *2C, KDM5C* and *6A* [55]. In a study involving 324 patients, approximately 15% of IMiD‐resistant cases exhibited no discernible genetic mutational drivers, prompting a strong speculation whether transcriptional regulation driven by epigenetic changes in the chromatin organisation underwrites their resistant biology [18]. As this mechanism is both reversible and potentially targetable, it is crucial to understand the epigenetic basis of IMiD resistance to develop effective resensitisation strategies.

**Fig. 3:**
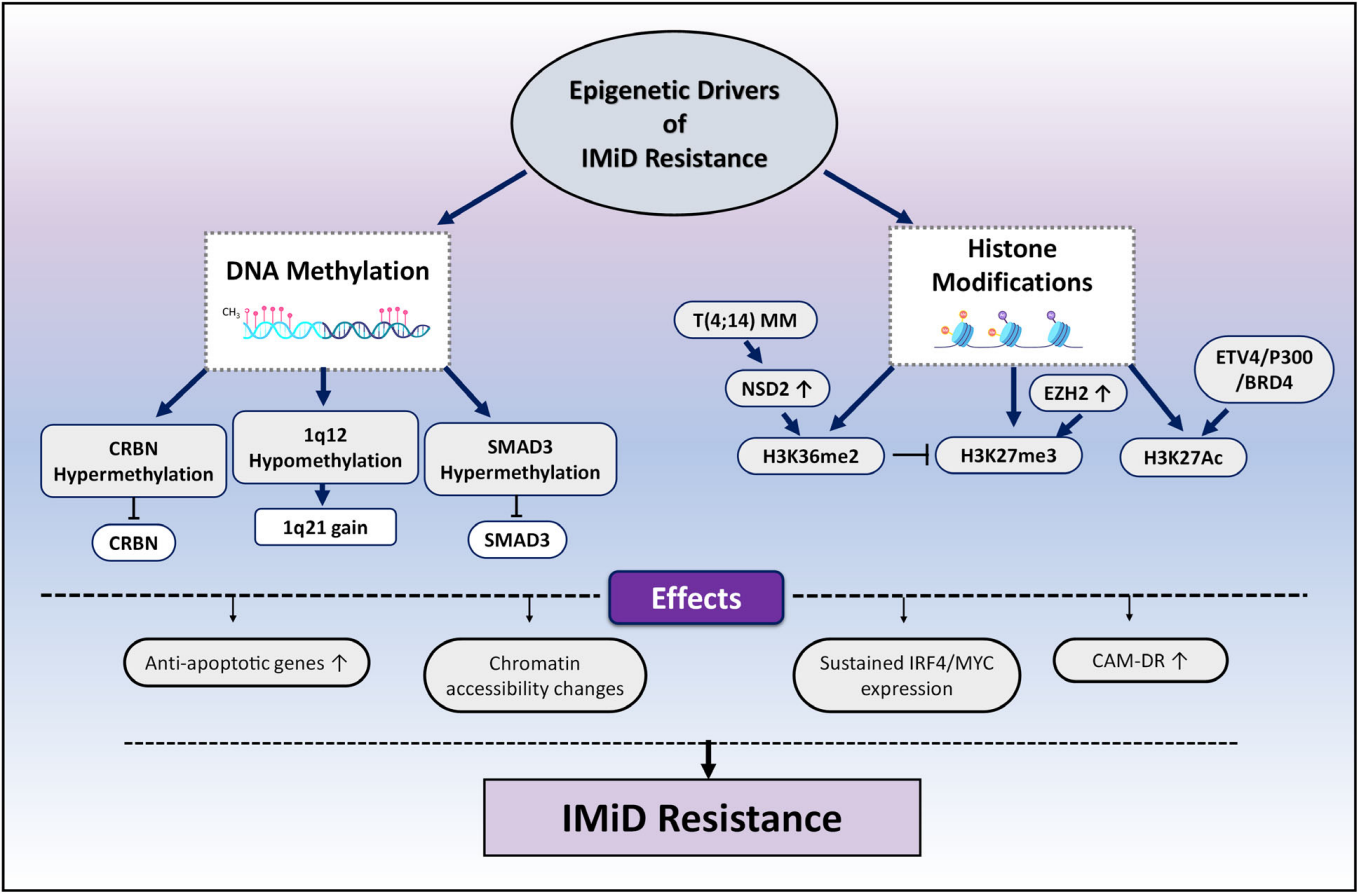
Epigenetic drivers of drug resistance in MM. DNA methylation and histone modifications are the critical epigenetic changes driving IMiD resistance. Among the DNA methylation related changes, hypermethylation of CRBN results in silencing of CRBN gene expression resulting in IMiD target reduction driven resistance. Also, hypomethylation of 1q12 contributes to 1q21 gain leading to the overexpression of many critical genes such as FAM72 and PBX1 that promote MM cell proliferation and IMiD resistance. Similarly, SMAD3 hypermethylation has been observed in many of the IMiD‐resistant cell models. Among the key histone modifications, H3K36me2 is driven by high NSD2 (histone methyltransferase) expression due to translocation (4;14), which is a frequent event in MM, leading to poor prognosis. Also, overexpression of EZH2 drives an increase in H3K27me3 levels, which leads to the silencing of many tumour suppressor genes. Notably, H3K36me2 has an antagonising relationship with H3K27me2. In addition, IMiD‐resistant cells maintain P300/BRD4 super‐enhancer occupancy on IKAROS/AIOLOS when cobound by ETV4, leading to sustained expression of IRF4/c‐MYC. P300/BRD4 is a critical regulator of H3K27Ac, which drives active gene expression. Overall, these above‐mentioned epigenetic changes along with others can drive chromatin accessibility changes, leading to upregulation of anti‐apoptotic genes, downregulation of tumour suppressor genes, sustained expression of MM driver genes such as IRF4/c‐MYC and upregulation of genes responsible for increased adhesion between MM cells and BMSC contributing to IMiD resistance. BRD4, Bromodomain 4; CAM‐DR, Cell adhesion‐mediated drug resistance; CRBN, cereblon; ETV4, E‐twenty‐six (ETS) variant transcription factor 4; EZH2, Enhancer of zeste homologue 2; FAM72, Family with sequence similarity 72; H3K27Ac, histone H3 lysine 27 acetylation; H3K27me3, histone H3 lysine 27 trimethylation; H3K36me2, histone H3 lysine 36 dimethylation; NSD2, Nuclear Receptor Binding SET Domain Protein 2; P300, Histone acetyltransferase p300; IMiD, immunomodulatory drug; IRF4, Interferon regulatory factor 4; PBX1, pre‐B‐cell leukaemia transcription factor 1; SMAD3, Mothers against decapentaplegic homologue 3; t(4;14), translocation (4;14).

The epigenetic drivers of IMiD resistance are summarised in Fig. 3. Hypermethylation of the *CRBN* enhancer region resulting in the silencing of CRBN has been linked to IMiD resistance, with an increased differential hypermethylation in IMiD‐refractory cases compared to healthy plasma cells [56]. A phase 1b study on 18 heavily pretreated RRMM patients (Len/Pom refractory), azacytidine which is a hypomethylating agent when combined with LEN and DEX showed an overall clinical benefit of 50% [57].

Hypomethylation of 1q12 has been shown to amplify the genomic regions juxtaposed to it leading to 1q21 copy number gains [58]. 1q gain was a key genetic driver of lenalidomide and pomalidomide resistance in RRMM gene analysis [18]. Around 25% of MM‐specific differentially methylated genes lie within the 1q21.1 locus, including *FAM72*, which promotes myeloma cell proliferation via FOXM1 activation following promoter demethylation [59]. Epigenetic activation of *PBX1*, another 1q gene, enhances FOXM1‐driven transcriptional programmes linked to poor prognosis [60]. In the same study, *EZH2* mutation was identified as a novel driver of IMiD‐resistant clones.

In preclinical resistant cell line model, transcriptomic profiling indicated that the resistant phenotype was largely driven by widespread gene downregulation through DNA methylation, with SMAD3 emerging as a consistently suppressed gene across resistant lines. Importantly, treatment with a combination of 5‐azacytidine and an EZH2 inhibitor restored chromatin accessibility, reactivated SMAD3 expression and significantly resensitised resistant cells to both lenalidomide and pomalidomide [61].

Histone modifications, particularly those affecting histone H3, play a critical role in mediating epigenetic drug resistance. Methylation at lysine residues 4 and 36 (H3K4 and H3K36), as well as at lysine 27 and 9 (H3K27 and H3K9), have been prominently implicated in these mechanisms [62].

H3K27me3, a key repressive histone mark catalysed by EZH2, has been previously implicated in CAM‐DR‐mediated IMiD resistance. Akt‐mediated inactivating phosphorylation of EZH2, driven by the bone marrow microenvironment and IL‐6 signalling, leads to reduced H3K27me3, sustaining the expression of anti‐apoptotic genes such as *IGF1, BCL2* and *HIF1α* [63, 64].

Analysis of large clinical trial datasets involving nearly 1500 myeloma patients has revealed that high EZH2 expression is independently linked to poor outcomes [65]. Targeting EZH2 with selective small molecule inhibitors in myeloma cell lines and patient samples led to cell cycle arrest and apoptosis, supporting its role as a promising therapeutic target, including in high‐risk disease. Mechanistically, EZH2 inhibition upregulated CDK inhibitors such as *p21* and *p15*, genes normally repressed by H3K27me3 [65].

IMiD resistance can also be mediated by pathways involving ETV4/p300/BRD4 axis. In resistant myeloma cells, MYC and IRF4 expression are sustained by the binding of transcription factors such as ETV4 and BATF, which recruit p300 and BRD4 [66, 67, 68]. High ETV4 expression correlated with shorter PFS and OS in CoMMpass patients receiving IMiDs and in POLLUX trial patients treated with lenalidomide and dexamethasone [4, 66].

In addition, emerging evidence has identified NCOR2, a chromatin remodelling factor, as a key contributor to multidrug resistance in MM, including resistance to IMiDs. NCOR2 was shown to form a repressive complex with the nucleosome remodelling and deacetylase (NuRD) complex, which binds directly to the CD180 promoter and suppresses its transcription, resulting in a reduction in MYC [69]. IMiD‐resistant cell models showed a downregulation in NCOR2 and CD180, with a subsequent upregulation in MYC [69].

### Overcoming IMiD resistance in the clinic

**Fig. 4:**
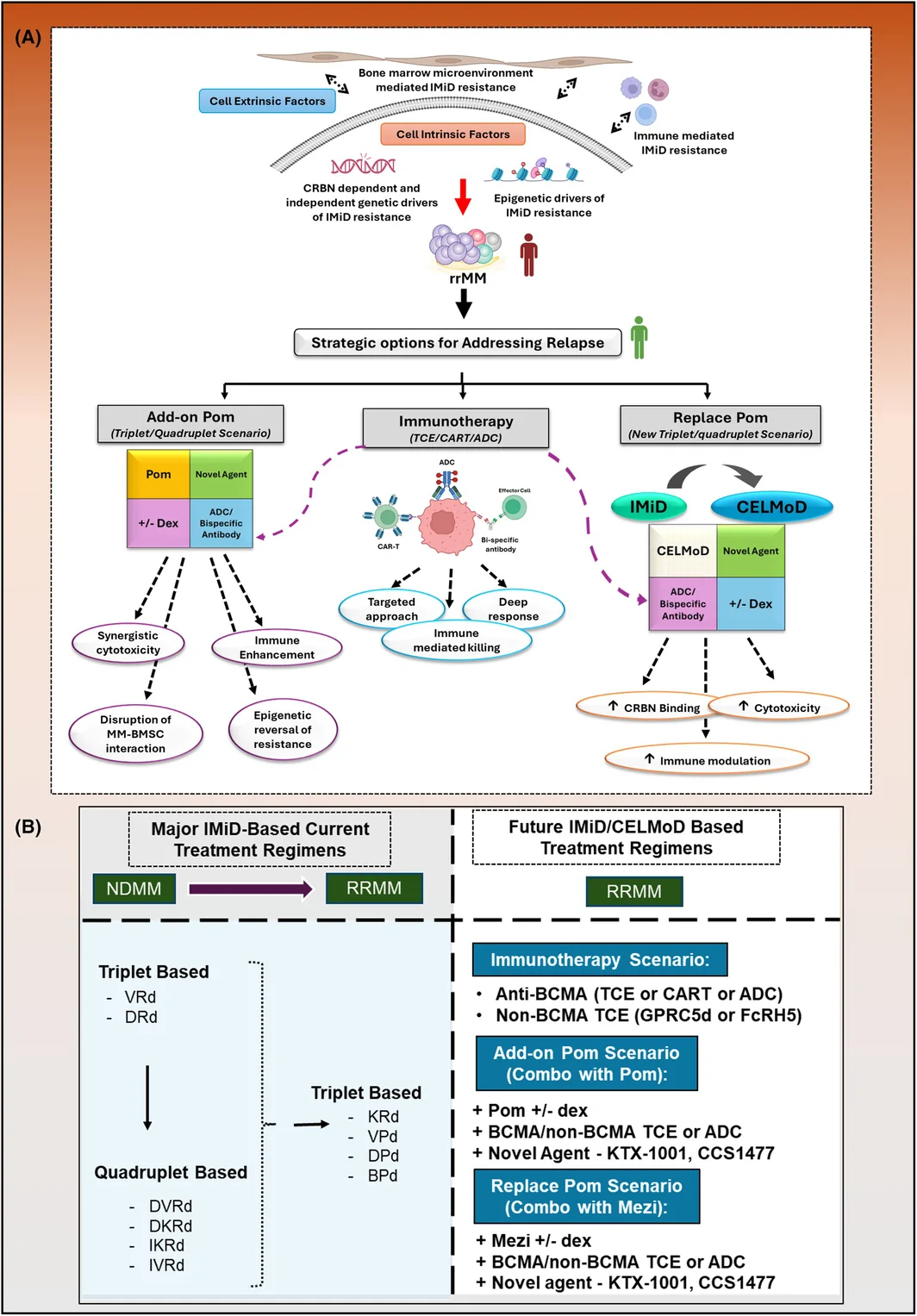
Current and emerging strategies to overcome resistance to IMiD and IMiD‐based regimens in relapsed myeloma. (A) Sources of resistance and therapeutic strategies in relapse. Various cell intrinsic genetic and epigenetic factors as well as cell extrinsic factors such as adhesion to bone marrow microenvironment and immune‐mediated factors drive IMiD resistance in MM. The current therapeutic strategies for reversing resistance are (i) immunotherapy using bispecific antibody or CAR T or ADC (ii) combination triplet‐ Add‐on to Pom or Quadruplet where a combination of (A) Pom, (B) a novel agent (e.g. epigenetic resensitiser) that can reverse/reprogramme resistance mechanisms, (c) Immunotherapy agents such as ADC (e.g. belantamab) or TCE that can synergise and enhance cell killing (D) with or without dexamethasone. Collectively, these strategies address RRMM through a multifaceted therapeutic approach. (iii) Replace‐Pom (Triplet combination) where Pom can be replaced with a more potent drug such as a Mezigdomide in combination with approved agents as in (ii). (B) Potential combination approaches for approved agents in relapse with IMiDs and CELMoDs. Various combinations in triplet and quadruplet regimens will establish key ‘winning’ combinations based on safety and efficacy data. ADC, antibody–drug conjugate; BCMA, B‐cell maturation antigen; BMSC, bone marrow stromal cell; BPd, belantamab mafodotin–pomalidomide–dexamethasone; CAR‐T, chimeric antigen receptor T cell; CBP, CREB‐binding protein; CELMoD, cereblon E3 ligase modulator; CRBN, cereblon; Dex, dexamethasone; DPd, daratumumab–pomalidomide–dexamethasone; DRd, daratumumab–lenalidomide–dexamethasone; DVd, daratumumab–bortezomib–dexamethasone; DVRd, daratumumab–bortezomib–lenalidomide–dexamethasone; DKRd, daratumumab–carfilzomib–lenalidomide–dexamethasone; EP300, E1A‐binding protein p300; FcRH5, Fc receptor‐homologue 5; GPRC5D, G protein‐coupled receptor class C group 5 member D; IMiD, immunomodulatory drug; IKRd, isatuximab–carfilzomib–lenalidomide–dexamethasone; IVRd, isatuximab–bortezomib–lenalidomide‐dexamethasone; KRd, carfilzomib–lenalidomide–dexamethasone; MM, multiple myeloma; NDMM, newly diagnosed multiple myeloma; NK, natural killer; Pom, pomalidomide; RRMM, relapsed/refractory multiple myeloma; TCE, T‐cell engager; TCR, T‐cell receptor; VRd, bortezomib–lenalidomide–dexamethasone; VPd, bortezomib–pomalidomide–dexamethasone.

As elaborated above, many cell intrinsic and extrinsic mechanisms drive clinical resistance to IMiD and IMiD‐based combination therapy in myeloma patients. As a result, when patients are exposed to frontline multiagent therapy and subsequently relapse, both the tumour and its resident tumour microenvironment are significantly altered. To address clinical resistance to IMiDs and IMiD‐based combination regimens, we must appreciate the evolving therapeutic landscape. The current options to therapeutically overcome IMiD resistance and some potential clinical combinations that could be considered are summarised in Fig. 4A,B, respectively.

Myeloma care is rapidly changing worldwide with the expanding use of the triplet and adoption of quadruplet‐based regimens in NDMM (Fig. 4B). Triplets such as daratumumab (D)‐lenalidomide (R)‐dexamethasone (d) [DRd] or lenalidomide (R)‐bortezomib (V)‐dexamethasone (d) [RVd] are established and routinely used followed by transplant and lenalidomide maintenance in transplant‐eligible patients. Notably, RRMM patients treated with the triplet regimen of daratumumab, pomalidomide and low‐dose dexamethasone showed a coordinated immune response whereby the depletion of NK and B cells by daratumumab treatment was counterbalanced by increased proliferation and activation of NK and CD8+ T cells along with a shift from naïve to effector memory T cells induced by pomalidomide treatment [34]. This combination showed similar and potent immunostimulatory effects in len‐refractory and nonrefractory patient subgroups [34].

More recently, clinical studies of quadruplet‐based regimens using DVRd (PERSEUS study), Isatuximab (Isa)‐Carfilzomib (K)‐Rd [Isa‐KRd] (MIDAS study), and Isa‐VRd (IMROZ study) have improved clinical outcomes with estimated median PFS between 100 and 200 months (median not reached) across these regimens from frontline therapy [70, 71, 72, 73]. These studies also demonstrate MRD‐guided and a time‐limited maintenance approach with lenalidomide and anti‐CD38 antibody as a possible new standard of care.

Developing effective therapeutic strategies against relapsed disease in these patients is critical to improving outcomes. Immunotherapies as a class are emerging as a core component of myeloma care in relapse and there are multiple options based on new target and therapeutic modalities to engage these targets (Fig. 4A). Both monotherapy and combinations of immunotherapeutic approaches against these targets are currently reporting improved clinical outcomes in comparison to historical standard of care. Immunotherapeutic target choice is a key area of research and BCMA, GPRC5D or FcRH5 are competitive in this space. Following target selection (e.g. BCMA), currently it is crucial to consider what modality of single agent therapy is to be selected between bispecific T‐cell engagers or CAR T. Understanding the benefit of properly sequencing the anti‐BCMA therapies would be crucial, especially for CAR T‐based therapies. Alternatively, combination therapies are also a current option for anti‐BCMA‐ADC. In the United Kingdom, belantamab mafodotin has received approval in combination with both pomalidomide‐dex and bortezomib‐dex. For the T‐cell engagers, it is reasonable to pair them with immunomodulatory drugs over proteasome inhibitors due to established mechanistic synergy. Trials are underway combining T‐cell engagers with IMiDs or CELMoDs such as the phase I trial (NCT06348108) evaluating the combination of talquetamab (Tal) with iberdomide and dex in RRMM patients, MonumenTAL‐6 phase III trial (NCT06208150) comparing Tal‐pomalidomide combination with Tal‐teclistamab, OPTIMMAL trial (NCT06461988) evaluating the combination of Tal‐lenalidomide as maintenance therapy [74].

Data available to date do not clarify if CELMoD (Iberdomide and Mezigdomide) will be clinically effective to replace pomalidomide as part of new triplet combinations in early relapse. Mezigdomide and Iberdomide demonstrate significantly stronger binding to CRBN than earlier IMiDs, with an IC₅₀ of 0.03 μm and 0.06 μm, respectively, compared to 1.5 μm and 1.2 μm for lenalidomide and pomalidomide [5]. This > 20‐fold higher affinity results in faster degradation of CRBN substrates, as shown by the more rapid protein reduction observed with 0.1 μm Iberdomide versus 1 μm pomalidomide. Both mezigdomide and iberdomide have demonstrated clinical activity in heavily pretreated RRMM, including patients with triple‐class refractory disease who had previously received lenalidomide, pomalidomide, proteasome inhibitor or anti‐CD38 therapies [54, 75]. The enhanced activity may be attributed to both greater CRBN‐binding strength and potentially more efficient recruitment and function of the E3 ubiquitin ligase complex.

In the CC‐220‐MM‐001 trial, Iberdomide induced strong substrate protein degradation even in IMiD‐refractory patients and those with low CRBN levels. Immunohistochemical analysis of 114 bone marrow biopsies showed that CRBN remained detectable in nearly all CD138^+^ cells even in patients refractory to one or both IMiDs. No significant differences in CRBN or Aiolos expression were observed based on IMiD‐refractory status, triple‐class resistance or prior lines of therapy, though a slight decrease in CRBN was noted in patients recently treated with pomalidomide. Responders with CRBN genetic alterations had similar progression‐free survival (10.9 months) and duration of response (9.5 months) to those without such alterations [15].

Mezigdomide, the most potent drug of the class, has shown superior clinical activity in Phase I trials and could be a preferred choice to combine with other agents [54]. Interestingly, Mezigdomide is being tested in combination with Carfilzomib/Bortezomib and dexamethasone in RRMM patients who had more than one line of therapy and are lenalidomide resistant (SUCCESSOR 1 and 2 trials) [76, 77]. If approved, this combination will provide an option to replace pomalidomide in early relapse. Further therapeutic options on the improvement on the T‐cell engagers or CAR Ts would be to add a CELMoD in combination with other novel agents. For belantamab mafodotin, adding novel combinations including CELMoDs would be logical, to improve upon currently licenced therapeutic combinations.

In parallel with advances in CELMoDs and immune‐based combinations, increasing attention has focused on biomarker‐driven strategies, particularly the exploitation of BCL‐2 dependence in t(11;14) multiple myeloma using venetoclax. The t(11;14)(q13;q32) translocation, which occurs in approximately 15–20% of multiple myeloma (MM) cases, identifies a biologically distinct subgroup marked by elevated BCL‐2 expression and increased reliance on BCL‐2‐dependent anti‐apoptotic signalling [78]. Consistent with this biology, venetoclax has demonstrated preferential activity in t(11;14) relapsed/refractory MM (RRMM), including as monotherapy in a cohort of 30 patients (including those who were double refractory to lenalidomide and bortezomib) where an overall response rate (ORR) of 40% was observed, significantly exceeding responses in non‐t(11;14) patients (*n* = 36) [79]. Enhanced efficacy has been reported with combination approaches: in the phase I study NCT03314181, venetoclax plus daratumumab and dexamethasone (VenDd) in t(11;14) RRMM and venetoclax plus bortezomib (VenDVd) in cytogenetically unselected RRMM achieved ORRs of 96% (all ≥VGPR) and 92% (79% ≥ VGPR), respectively, further supporting the clinical activity of venetoclax‐based combinations, particularly in t(11;14) disease [80]. These findings are reinforced by a large retrospective analysis of 232 MM patients treated at the Mayo Clinic between January 2015 and December 2023, in which t(11;14) patients (n = 190) achieved an ORR of 64% and a median PFS of 11.8 months; notably, inferior PFS was associated with the presence of additional high‐risk cytogenetic abnormalities [81]. Importantly, the majority of patients in this real‐world cohort were refractory to lenalidomide (75%) and pomalidomide (61%), underscoring the activity of venetoclax in heavily pretreated and IMiD‐refractory populations [81]. Sonrotoclax (BGB‐11417) is a next‐generation, highly selective and more potent BCL‐2 inhibitor than venetoclax. Preliminary data suggest encouraging activity, with the sonrotoclax plus carfilzomib and dexamethasone cohort (BGB‐11417‐105) achieving an 84% ORR and a 32% CR/sCR rate in heavily pretreated patients with t(11;14) relapsed/refractory multiple myeloma [82].

Given the role of cullin neddylation and COP9‐mediated regulation of CRL4^CRBN^, inhibition of the neddylation pathway has been explored as a potential therapeutic strategy in multiple myeloma. Preclinical studies have shown that the NEDD8‐activating enzyme inhibitor pevonedistat (MLN4924) can enhance NK cell‐mediated cytotoxicity by increasing surface expression of the NKG2D ligands MICA and MICB on myeloma cells [83]. In this context, neddylation inhibition may cooperate with IMiDs via increased CRBN expression to further augment MICA upregulation. However, while these findings highlight an immunomodulatory role for neddylation targeting, early phase clinical trials on pevonedistat demonstrate modest clinical activity and its clinical significance in IMiD‐resistant multiple myeloma has yet to be defined [84].

In addition, as an add‐on approach, trials looking for mechanisms for resensitisation to IMiD have described the use of epigenetic drugs as a possible alternative. After culturing lenalidomide‐resistant (−LR) and pomalidomide‐resistant (‐PR) human myeloma cell lines, Dimopoulos et al showed that after exposure of these cell lines to 5‐azacytidine and EPZ‐6438, chromatin accessibility changes and expression of SMAD3 were restored, and resensitisation of cells to both lenalidomide and pomalidomide was observed [61]. Also, EZH2 represents a compelling therapeutic target, and several studies are currently investigating its inhibition either as monotherapy or in combination with established agents such as bortezomib or lenalidomide [61].

Among the novel agents and mechanisms, two epigenetic compounds in the clinic are noteworthy. KTX‐1001, a selective NSD2 inhibitor currently in clinical trials, represents a promising strategy to overcome IMiD resistance in NSD2^high^ and t(4;14)‐positive relapsed and refractory MM [85]. t(4;14) chromosomal translocation is found in approximately 15–19% of MM cases. This translocation juxtaposes the *NSD2* gene encoding a histone methyltransferase specific for H3K36 near a potent super‐enhancer region, resulting in its overexpression. Elevated NSD2 activity promotes transcriptional changes that enhance cell proliferation and adhesion, thereby contributing to a drug‐resistant phenotype [86]. The t(4;14) translocation in MM has been associated with early relapse following immunomodulatory drug (IMiD) therapy, highlighting its role in promoting treatment resistance [4]. Functional studies have demonstrated that *NSD2* knockdown in MM cell lines significantly impairs their ability to adhere to the extracellular matrix (ECM) and leads to a marked reduction in clonogenicity and proliferation [87]. In preclinical resistant models, KTX1001 shows potent anti‐adhesive effects resulting in increased sensitivity to anticancer agents [88]. These findings underscore the therapeutic potential of targeting NSD2‐driven epigenetic alterations. Notably, mechanism driven combination of KTX1001 with belantamab and pomalidomide in high NSD2 or t(4;14) lenalidomide‐refractory patients could be a potential approach.

Additionally, and as previously discussed, the ETV4/p300/BRD4 axis mediated upregulation of MYC and IRF4 plays a crucial role in IMiD resistance and because of this, there is interest in exploring ways to block these complementary pathways. One promising approach is using epigenetic agents such as Inobrodib (CCS1477), which blocks p300 and its partner protein CBP. Inobrodib has shown strong effects against myeloma cells and works even better when combined with IMiDs like pomalidomide, offering a potential new strategy to overcome IMiD resistance [89, 90]. SGC‐CBP30, a CBP/EP300 bromodomain inhibitor which targets the IRF4/c‐Myc axis resensitised MM cells to lenalidomide, effectively restoring drug response [19]. Notably, SGC‐CBP30 also reactivated lenalidomide sensitivity in two additional resistant cell lines with low but detectable CRBN levels, indicating a therapeutic strategy where CRBN is present but functionally insufficient, demonstrating scientific rationale for developing p300 inhibitors in IMiD‐resistant myeloma [19]. These novel agents can potentially open an alternative quadruplet approach replacing CD38 which is used frontline with new immunotherapeutic targets (BCMA, GPRC5D and FcRH5).

As such, the combination of (i) immunotherapeutic agents targeting surface membrane proteins (BCMA, GPRC5D and FcRH5) (ii) IMiD (pomalidomide) (iii) an IMiD resensitising novel agent with or without (iv) dexamethasone has robust strategic rationale for clinical testing. In this context, novel therapies such as the NSD2 inhibitor or CBP/EP300 inhibitor which have the potential to resensitise myeloma cells to immunomodulatory agents hold significant promise and potentially could be used as the IMiD resensitising agent in the quadruplet therapeutic strategy. However, the current evidence is limited to preclinical studies and early phase clinical trials, and further clinical evaluation is required to establish efficacy and safety. Overall, in early relapse, therapeutically augmenting pomalidomide by adding novel agents to reverse IMiD‐resistant biology will open up a space for exploring cost effective, clinically competent treatment combinations. If successful, these novel agents could augment the licenced triplets in early relapse.

## Author contributions

MF and DN are listed as cofirst authors and contributed equally to this manuscript. MF and DN conceived the review, conducted the literature search, synthesised the data and wrote the initial draft of the manuscript. KR contributed to the writing of the clinical sections of the review and, together with AT, provided critical intellectual input and contributed to the interpretation of the literature. AT provided overall scientific supervision and critical guidance during the development, writing and revisions of the manuscript. All authors contributed to and approved the final version of the manuscript.

## Conflict of interest

AT is funded by research grants from K36 Therapeutics, Cell Centric and Oxford Translational Myeloma Centre. The other authors declare no conflict of interest.

## References

[1] Bjorklund CC , Kang J , Amatangelo M , Polonskaia A , Katz M , Chiu H , Couto S , Wang M , Ren Y , Ortiz M *et al*. (2020) Iberdomide (CC‐220) is a potent cereblon E3 ligase modulator with antitumor and immunostimulatory activities in lenalidomide‐ and pomalidomide‐resistant multiple myeloma cells with dysregulated CRBN. Leukemia 34, 1197–1201. 31719682 10.1038/s41375-019-0620-8 PMC7214241

[2] Fischer ES , Böhm K , Lydeard JR , Yang H , Stadler MB , Cavadini S , Nagel J , Serluca F , Acker V , Lingaraju GM *et al*. (2014) Structure of the DDB1‐CRBN E3 ubiquitin ligase in complex with thalidomide. Nature 512, 49–53. 25043012 10.1038/nature13527 PMC4423819

[3] Chen LY & Gooding S (2022) Tumor and microenvironmental mechanisms of resistance to immunomodulatory drugs in multiple myeloma. Front Oncol 12, 1038329. 36439455 10.3389/fonc.2022.1038329 PMC9682014

[4] Teoh PJ , Koh MY , Mitsiades C , Gooding S & Chng WJ (2025) Resistance to immunomodulatory drugs in multiple myeloma: the cereblon pathway and beyond. Haematologica 110, 1074–1091. 39569422 10.3324/haematol.2024.285636 PMC12050938

[5] Bird S & Pawlyn C (2023) IMiD resistance in multiple myeloma: current understanding of the underpinning biology and clinical impact. Blood 142, 131–140. 36929172 10.1182/blood.2023019637

[6] Thakurta A , Pierceall WE , Amatangelo MD , Flynt E & Agarwal A (2021) Developing next generation immunomodulatory drugs and their combinations in multiple myeloma. Oncotarget 12, 1555–1563. 34316334 10.18632/oncotarget.27973 PMC8310669

[7] Franssen LE , Nijhof IS , Couto S , Levin MD , Bos GMJ , Broijl A , Klein SK , Ren Y , Wang M , Koene HR *et al*. (2018) Cereblon loss and up‐regulation of c‐Myc are associated with lenalidomide resistance in multiple myeloma patients. Haematologica 103, e368–e371. 29545338 10.3324/haematol.2017.186601 PMC6068039

[8] Zhu YX , Braggio E , Shi CX , Bruins LA , Schmidt JE , van Wier S , Chang XB , Bjorklund CC , Fonseca R , Bergsagel PL *et al*. (2011) Cereblon expression is required for the antimyeloma activity of lenalidomide and pomalidomide. Blood 118, 4771–4779. 21860026 10.1182/blood-2011-05-356063 PMC3208291

[9] Akuffo AA , Alontaga AY , Metcalf R , Beatty MS , Becker A , McDaniel JM , Hesterberg RS , Goodheart WE , Gunawan S , Ayaz M *et al*. (2018) Ligand‐mediated protein degradation reveals functional conservation among sequence variants of the CUL4‐type E3 ligase substrate receptor cereblon. J Biol Chem 293, 6187–6200. 29449372 10.1074/jbc.M117.816868 PMC5912449

[10] Jones JR , Barber A , le Bihan YV , Weinhold N , Ashby C , Walker BA , Wardell CP , Wang H , Kaiser MF , Jackson GH *et al*. (2021) Mutations in CRBN and other cereblon pathway genes are infrequently associated with acquired resistance to immunomodulatory drugs. Leukemia 35, 3017–3020. 34373585 10.1038/s41375-021-01373-4 PMC8478640

[11] Gooding S , Ansari‐Pour N , Towfic F , Ortiz Estévez M , Chamberlain PP , Tsai KT , Flynt E , Hirst M , Rozelle D , Dhiman P *et al*. (2021) Multiple cereblon genetic changes are associated with acquired resistance to lenalidomide or pomalidomide in multiple myeloma. Blood 137, 232–237. 33443552 10.1182/blood.2020007081 PMC7893409

[12] Neri P , Maity R , Keats JJ , Tagoug I , Simms J , Auclair D , Lonial S & Bahlis NJ (2016) Cereblon splicing of exon 10 mediates IMiDs resistance in multiple myeloma: clinical validation in the CoMMpass trial. Blood 128, 120.27162243

[13] Zhu YX , Dutta S , Ahmann GJ , Bruins L , Arribas M , Chen X , Polavarapu A , Chapman J , Lopez I , Chesi M *et al*. (2025) A novel cereblon variant with both exon 8 and 10 deletions in newly diagnosed and relapsed multiple myeloma. Blood Neoplasia 2, 100099. 40612717 10.1016/j.bneo.2025.100099 PMC12221632

[14] Kortüm KM , Mai EK , Hanafiah NH , Shi CX , Zhu YX , Bruins L , Barrio S , Jedlowski P , Merz M , Xu J *et al*. (2016) Targeted sequencing of refractory myeloma reveals a high incidence of mutations in CRBN and Ras pathway genes. Blood 128, 1226–1233. 27458004 10.1182/blood-2016-02-698092 PMC5524534

[15] Amatangelo M , Flynt E , Stong N , Ray P , van Oekelen O , Wang M , Ortiz M , Maciag P , Peluso T , Parekh S *et al*. (2024) Pharmacodynamic changes in tumor and immune cells drive iberdomide's clinical mechanisms of activity in relapsed and refractory multiple myeloma. Cell Rep Med 5, 101571. 38776914 10.1016/j.xcrm.2024.101571 PMC11228401

[16] Dubiel W , Chaithongyot S , Dubiel D & Naumann M (2020) The COP9 signalosome: a multi‐DUB complex. Biomolecules 10, 1082. 32708147 10.3390/biom10071082 PMC7407660

[17] Gooding S , Ansari‐Pour N , Kazeroun M , Karagoz K , Polonskaia A , Salazar M , Fitzsimons E , Sirinukunwattana K , Chavda S , Ortiz Estevez M *et al*. (2022) Loss of COP9 signalosome genes at 2q37 is associated with IMiD resistance in multiple myeloma. Blood 140, 1816–1821. 35853156 10.1182/blood.2022015909 PMC10653034

[18] Ansari‐Pour N , Samur M , Flynt E , Gooding S , Towfic F , Stong N , Estevez MO , Mavrommatis K , Walker B , Morgan G *et al*. (2023) Whole‐genome analysis identifies novel drivers and high‐risk double‐hit events in relapsed/refractory myeloma. Blood 141, 620–633. 36223594 10.1182/blood.2022017010 PMC10163277

[19] Zhu YX , Shi CX , Bruins LA , Wang X , Riggs DL , Porter B , Ahmann JM , de Campos CB , Braggio E , Bergsagel PL *et al*. (2019) Identification of lenalidomide resistance pathways in myeloma and targeted resensitization using cereblon replacement, inhibition of STAT3 or targeting of IRF4. Blood Cancer J 9, 19. 30741931 10.1038/s41408-019-0173-0 PMC6370766

[20] Ocio EM , Fernández‐Lázaro D , San‐Segundo L , López‐Corral L , Corchete LA , Gutiérrez NC , Garayoa M , Paíno T , García‐Gómez A , Delgado M *et al*. (2015) In vivo murine model of acquired resistance in myeloma reveals differential mechanisms for lenalidomide and pomalidomide in combination with dexamethasone. Leukemia 29, 705–714. 25102946 10.1038/leu.2014.238

[21] Ng YLD , Ramberger E , Bohl SR , Dolnik A , Steinebach C , Conrad T , Müller S , Popp O , Kull M , Haji M *et al*. (2022) Proteomic profiling reveals CDK6 upregulation as a targetable resistance mechanism for lenalidomide in multiple myeloma. Nat Commun 13, 1009. 35197447 10.1038/s41467-022-28515-1 PMC8866544

[22] Zhou N , Gutierrez‐Uzquiza A , Zheng XY , Chang R , Vogl DT , Garfall AL , Bernabei L , Saraf A , Florens L , Washburn MP *et al*. (2019) RUNX proteins desensitize multiple myeloma to lenalidomide via protecting IKZFs from degradation. Leukemia 33, 2006–2021. 30760870 10.1038/s41375-019-0403-2 PMC6687534

[23] Bjorklund CC , Ma W , Wang ZQ , Davis RE , Kuhn DJ , Kornblau SM , Wang M , Shah JJ & Orlowski RZ (2011) Evidence of a role for activation of Wnt/beta‐catenin signaling in the resistance of plasma cells to lenalidomide. J Biol Chem 286, 11009–11020. 21189262 10.1074/jbc.M110.180208 PMC3064156

[24] Bjorklund CC , Baladandayuthapani V , Lin HY , Jones RJ , Kuiatse I , Wang H , Yang J , Shah JJ , Thomas SK , Wang M *et al*. (2014) Evidence of a role for CD44 and cell adhesion in mediating resistance to lenalidomide in multiple myeloma: therapeutic implications. Leukemia 28, 373–383. 23760401 10.1038/leu.2013.174 PMC3874423

[25] Dhodapkar MV (2024) Immune status and selection of patients for immunotherapy in myeloma: a proposal. Blood Adv 8, 2424–2432. 38564776 10.1182/bloodadvances.2023011242 PMC11112605

[26] Russell BM & Avigan DE (2023) Immune dysregulation in multiple myeloma: the current and future role of cell‐based immunotherapy. Int J Hematol 117, 652–659. 36964840 10.1007/s12185-023-03579-x PMC10039687

[27] Prabhala R , Pierceall WE , Samur M , Potluri LB , Hong K , Peluso T , Talluri S , Wang A , Katiki A , Vangala SD *et al*. (2023) Immunomodulation of NK, NKT and B/T cell subtypes in relapsed/refractory multiple myeloma patients treated with pomalidomide along with velcade and dexamethasone and its association with improved progression‐free survival. Front Oncol 13, 1271807. 38111533 10.3389/fonc.2023.1271807 PMC10726115

[28] Díaz‐Tejedor A , Lorenzo‐Mohamed M , Puig N , García‐Sanz R , Mateos MV , Garayoa M & Paíno T (2021) Immune system alterations in multiple myeloma: molecular mechanisms and therapeutic strategies to reverse immunosuppression. Cancers (Basel) 13, 1353. 33802806 10.3390/cancers13061353 PMC8002455

[29] Ubieto AJ , Martinez‐Lopez J , Paiva B , Rosiñol L , Gonzalez‐Medina J , Puig N , Cedena T , Calasanz MJ , Ramos ML , Alonso R *et al*. (2019) PS1351 critical analysis of the stringent complete response in multiple myeloma. An updated of the analysis based on the PETHEMA/GEM2012MENOS65 phase iii clinical trial. HemaSphere 3, 617.

[30] Pessoa de Magalhães RJ , Vidriales MB , Paiva B , Fernandez‐Gimenez C , García‐Sanz R , Mateos MV , Gutierrez NC , Lecrevisse Q , Blanco JF , Hernández J *et al*. (2013) Analysis of the immune system of multiple myeloma patients achieving long‐term disease control by multidimensional flow cytometry. Haematologica 98, 79–86. 22773604 10.3324/haematol.2012.067272 PMC3533663

[31] Prabhala RH , Pelluru D , Fulciniti M , Prabhala HK , Nanjappa P , Song W , Pai C , Amin S , Tai YT , Richardson PG *et al*. (2010) Elevated IL‐17 produced by TH17 cells promotes myeloma cell growth and inhibits immune function in multiple myeloma. Blood 115, 5385–5392. 20395418 10.1182/blood-2009-10-246660 PMC2902136

[32] Zavidij O , Haradhvala NJ , Mouhieddine TH , Sklavenitis‐Pistofidis R , Cai S , Reidy M , Rahmat M , Flaifel A , Ferland B , Su NK *et al*. (2020) Single‐cell RNA sequencing reveals compromised immune microenvironment in precursor stages of multiple myeloma. Nat Can 1, 493–506. 10.1038/s43018-020-0053-3 PMC7785110 33409501

[33] Görgün GT , Whitehill G , Anderson JL , Hideshima T , Maguire C , Laubach J , Raje N , Munshi NC , Richardson PG & Anderson KC (2013) Tumor‐promoting immune‐suppressive myeloid‐derived suppressor cells in the multiple myeloma microenvironment in humans. Blood 121, 2975–2987. 23321256 10.1182/blood-2012-08-448548 PMC3624943

[34] Pierceall WE , Amatangelo MD , Bahlis NJ , Siegel DS , Rahman A , van Oekelen O , Neri P , Young M , Chung W , Serbina N *et al*. (2020) Immunomodulation in Pomalidomide, dexamethasone, and Daratumumab‐treated patients with relapsed/refractory multiple myeloma. Clin Cancer Res 26, 5895–5902. 32928795 10.1158/1078-0432.CCR-20-1781

[35] Salminen A , Kaarniranta K & Kauppinen A (2012) Inflammaging: disturbed interplay between autophagy and inflammasomes. Aging (Albany NY) 4, 166–175. 22411934 10.18632/aging.100444 PMC3348477

[36] Franceschi C *et al*. (2000) Inflamm‐aging. An evolutionary perspective on immunosenescence. Ann N Y Acad Sci 908, 244–254. 10911963 10.1111/j.1749-6632.2000.tb06651.x

[37] Visram A , Cook J & Warsame R (2021) Smoldering multiple myeloma: evolving diagnostic criteria and treatment strategies. Hematology Am Soc Hematol Educ Program 2021, 673–681. 34889380 10.1182/hematology.2021000304 PMC8791169

[38] Gabrilovich DI & Nagaraj S (2009) Myeloid‐derived suppressor cells as regulators of the immune system. Nat Rev Immunol 9, 162–174. 19197294 10.1038/nri2506 PMC2828349

[39] Solimando AG , Malerba E , Leone P , Prete M , Terragna C , Cavo M & Racanelli V (2022) Drug resistance in multiple myeloma: soldiers and weapons in the bone marrow niche. Front Oncol 12, 973836. 36212502 10.3389/fonc.2022.973836 PMC9533079

[40] Giallongo C , Tibullo D , Parrinello NL , la Cava P , di Rosa M , Bramanti V , di Raimondo C , Conticello C , Chiarenza A , Palumbo GA *et al*. (2016) Granulocyte‐like myeloid derived suppressor cells (G‐MDSC) are increased in multiple myeloma and are driven by dysfunctional mesenchymal stem cells (MSC). Oncotarget 7, 85764–85775. 26967390 10.18632/oncotarget.7969 PMC5349872

[41] Kuwahara‐Ota S , Shimura Y , Steinebach C , Isa R , Yamaguchi J , Nishiyama D , Fujibayashi Y , Takimoto‐Shimomura T , Mizuno Y , Matsumura‐Kimoto Y *et al*. (2020) Lenalidomide and pomalidomide potently interfere with induction of myeloid‐derived suppressor cells in multiple myeloma. Br J Haematol 191, 784–795. 32558939 10.1111/bjh.16881

[42] Busch A , Zeh D , Janzen V , Mügge LO , Wolf D , Fingerhut L , Hahn‐Ast C , Maurer O , Brossart P & von Lilienfeld‐Toal M (2014) Treatment with lenalidomide induces immunoactivating and counter‐regulatory immunosuppressive changes in myeloma patients. Clin Exp Immunol 177, 439–453. 24712857 10.1111/cei.12343 PMC4226595

[43] Wallin A & Larsson SC (2011) Body mass index and risk of multiple myeloma: a meta‐analysis of prospective studies. Eur J Cancer 47, 1606–1615. 21354783 10.1016/j.ejca.2011.01.020

[44] Choa R , Panaroni C , Bhatia R & Raje N (2023) It is worth the weight: obesity and the transition from monoclonal gammopathy of undetermined significance to multiple myeloma. Blood Adv 7, 5510–5523. 37493975 10.1182/bloodadvances.2023010822 PMC10515310

[45] Ochiai M , Fierstein S , XsSali F , DeVito N , Purkey LR , May R , Correa‐Medina A , Kelley M , Page TD & DeCicco‐Skinner K (2023) Unlocking drug resistance in multiple myeloma: adipocytes as modulators of treatment response. Cancers (Basel) 15, 4347. 37686623 10.3390/cancers15174347 PMC10486466

[46] Bullwinkle EM , Parker MD , Bonan NF , Falkenberg LG , Davison SP & DeCicco‐Skinner KL (2016) Adipocytes contribute to the growth and progression of multiple myeloma: unraveling obesity related differences in adipocyte signaling. Cancer Lett 380, 114–121. 27317873 10.1016/j.canlet.2016.06.010

[47] Liu Z , Xu J , He J , Liu H , Lin P , Wan X , Navone NM , Tong Q , Kwak LW , Orlowski RZ *et al*. (2015) Mature adipocytes in bone marrow protect myeloma cells against chemotherapy through autophagy activation. Oncotarget 6, 34329–34341. 26455377 10.18632/oncotarget.6020 PMC4741456

[48] Mittal D , Gubin MM , Schreiber RD & Smyth MJ (2014) New insights into cancer immunoediting and its three component phases — elimination, equilibrium and escape. Curr Opin Immunol 27, 16–25. 24531241 10.1016/j.coi.2014.01.004 PMC4388310

[49] Swamydas M , Murphy EV , Ignatz‐Hoover JJ , Malek E & Driscoll JJ (2022) Deciphering mechanisms of immune escape to inform immunotherapeutic strategies in multiple myeloma. J Hematol Oncol 15, 17. 35172851 10.1186/s13045-022-01234-2 PMC8848665

[50] Sehgal K , das R , Zhang L , Verma R , Deng Y , Kocoglu M , Vasquez J , Koduru S , Ren Y , Wang M *et al*. (2015) Clinical and pharmacodynamic analysis of pomalidomide dosing strategies in myeloma: impact of immune activation and cereblon targets. Blood 125, 4042–4051. 25869284 10.1182/blood-2014-11-611426 PMC4481593

[51] Landgren O , Hultcrantz M , Diamond B , Lesokhin AM , Mailankody S , Hassoun H , Tan C , Shah UA , Lu SX , Salcedo M *et al*. (2021) Safety and effectiveness of weekly Carfilzomib, Lenalidomide, dexamethasone, and Daratumumab combination therapy for patients with newly diagnosed multiple myeloma: the MANHATTAN nonrandomized clinical trial. JAMA Oncol 7, 862–868. 33856405 10.1001/jamaoncol.2021.0611 PMC8050789

[52] Maura F , Boyle EM , Coffey D , Maclachlan K , Gagler D , Diamond B , Ghamlouch H , Blaney P , Ziccheddu B , Cirrincione A *et al*. (2023) Genomic and immune signatures predict clinical outcome in newly diagnosed multiple myeloma treated with immunotherapy regimens. Nat Can 4, 1660–1674. 10.1038/s43018-023-00657-1 PMC12065606 37945755

[53] Chen LY , van Oekelen O , Amatangelo M , Chow TT , Upadhyaya B , Lagana A , Kelly G , Kim‐Schulze S , Aleman A , Kurtova A *et al*. (2023) Mezigdomide treatment in relapsed‐refractory myeloma patients shifts bone marrow NK and T cell populations from exhaustion to activation. Blood 142, 4686.

[54] Richardson PG , Trudel S , Popat R , Mateos MV , Vangsted AJ , Ramasamy K , Martinez‐Lopez J , Quach H , Orlowski RZ , Arnao M *et al*. (2023) Mezigdomide plus dexamethasone in relapsed and refractory multiple myeloma. N Engl J Med 389, 1009–1022. 37646702 10.1056/NEJMoa2303194

[55] Walker BA , Mavrommatis K , Wardell CP , Ashby TC , Bauer M , Davies FE , Rosenthal A , Wang H , Qu P , Hoering A *et al*. (2018) Identification of novel mutational drivers reveals oncogene dependencies in multiple myeloma. Blood 132, 587–597. 29884741 10.1182/blood-2018-03-840132 PMC6097138

[56] Haertle L , Barrio S , Munawar U , Han S , Zhou X , Vogt C , Fernández RA , Bittrich M , Ruiz‐Heredia Y , da Viá M *et al*. (2021) Cereblon enhancer methylation and IMiD resistance in multiple myeloma. Blood 138, 1721–1726. 34115836 10.1182/blood.2020010452 PMC8569411

[57] Kalff A , Khong T , Mithraprabhu S , Bergin K , Reynolds J , Bowen KM , Thakurta A , Guzman R , Wang M , Couto S *et al*. (2019) Oral azacitidine (CC‐486) in combination with lenalidomide and dexamethasone in advanced, lenalidomide‐refractory multiple myeloma (ROAR study). Leuk Lymphoma 60, 2143–2151. 31184224 10.1080/10428194.2019.1571201

[58] Sawyer JR , Tian E , Heuck CJ , Johann DJ , Epstein J , Swanson CM , Lukacs JL , Binz RL , Johnson M , Sammartino G *et al*. (2015) Evidence of an epigenetic origin for high‐risk 1q21 copy number aberrations in multiple myeloma. Blood 125, 3756–3759. 25943786 10.1182/blood-2015-03-632075 PMC4463736

[59] Chatonnet F , Pignarre A , Sérandour AA , Caron G , Avner S , Robert N , Kassambara A , Laurent A , Bizot M , Agirre X *et al*. (2020) The hydroxymethylome of multiple myeloma identifies FAM72D as a 1q21 marker linked to proliferation. Haematologica 105, 774–783. 31221779 10.3324/haematol.2019.222133 PMC7049362

[60] Trasanidis N , Katsarou A , Ponnusamy K , Shen YA , Kostopoulos IV , Bergonia B , Keren K , Reema P , Xiao X , Szydlo RM *et al*. (2022) Systems medicine dissection of chr1q‐amp reveals a novel PBX1‐FOXM1 axis for targeted therapy in multiple myeloma. Blood 139, 1939–1953. 35015835 10.1182/blood.2021014391 PMC13127299

[61] Dimopoulos K , Søgaard Helbo A , Fibiger Munch‐Petersen H , Sjö L , Christensen J , Sommer Kristensen L , Asmar F , Hermansen NEU , O'Connel C , Gimsing P *et al*. (2018) Dual inhibition of DNMTs and EZH2 can overcome both intrinsic and acquired resistance of myeloma cells to IMiDs in a cereblon‐independent manner. Mol Oncol 12, 180–195. 29130642 10.1002/1878-0261.12157 PMC5792743

[62] Greer EL & Shi Y (2012) Histone methylation: a dynamic mark in health, disease and inheritance. Nat Rev Genet 13, 343–357. 22473383 10.1038/nrg3173 PMC4073795

[63] Kikuchi J , Koyama D , Wada T , Izumi T , Hofgaard PO , Bogen B & Furukawa Y (2015) Phosphorylation‐mediated EZH2 inactivation promotes drug resistance in multiple myeloma. J Clin Invest 125, 4375–4390. 26517694 10.1172/JCI80325 PMC4665777

[64] Croonquist PA & Van Ness B (2005) The polycomb group protein enhancer of zeste homolog 2 (EZH 2) is an oncogene that influences myeloma cell growth and the mutant ras phenotype. Oncogene 24, 6269–6280. 16007202 10.1038/sj.onc.1208771

[65] Pawlyn C , Bright MD , Buros AF , Stein CK , Walters Z , Aronson LI , Mirabella F , Jones JR , Kaiser MF , Walker BA *et al*. (2017) Overexpression of EZH2 in multiple myeloma is associated with poor prognosis and dysregulation of cell cycle control. Blood Cancer J 7, e549. 28362441 10.1038/bcj.2017.27 PMC5380911

[66] Neri P , Barwick BG , Jung D , Patton JC , Maity R , Tagoug I , Stein CK , Tilmont R , Leblay N , Ahn S *et al*. (2024) ETV4‐dependent transcriptional plasticity maintains MYC expression and results in IMiD resistance in multiple myeloma. Blood Cancer Discov 5, 56–73. 37934799 10.1158/2643-3230.BCD-23-0061 PMC10772538

[67] Yun S & Cleveland JL (2024) Transcriptional plasticity drives IMiD and p300 inhibitor resistance in multiple myeloma. Blood Cancer Discov 5, 5–7. 38085608 10.1158/2643-3230.BCD-23-0223 PMC10772544

[68] Welsh SJ , Barwick BG , Meermeier EW , Riggs DL , Shi CX , Zhu YX , Sharik ME , du MT , Abrego Rocha LD , Garbitt VM *et al*. (2024) Transcriptional heterogeneity overcomes super‐enhancer disrupting drug combinations in multiple myeloma. Blood Cancer Discov 5, 34–55. 37767768 10.1158/2643-3230.BCD-23-0062 PMC10772542

[69] Mori T , Verma R , Nakamoto‐Matsubara R , Siu KT , Panaroni C , Fulzele KS , Mukaihara K , Onyewadume C , Maebius A , Kato H *et al*. (2021) Low NCOR2 levels in multiple myeloma patients drive multidrug resistance via MYC upregulation. Blood Cancer J 11, 194. 34864816 10.1038/s41408-021-00589-y PMC8643354

[70] Sonneveld P , Dimopoulos MA , Boccadoro M , Quach H , Ho PJ , Beksac M , Hulin C , Antonioli E , Leleu X , Mangiacavalli S *et al*. (2024) Daratumumab, Bortezomib, Lenalidomide, and dexamethasone for multiple myeloma. N Engl J Med 390, 301–313. 38084760 10.1056/NEJMoa2312054

[71] Perrot A , Lambert J , Hulin C , Pieragostini A , Karlin L , Arnulf B , Rey P , Garderet L , Macro M , Escoffre‐Barbe M *et al*. (2025) Measurable residual disease–guided therapy in newly diagnosed myeloma. N Engl J Med 393, 425–437. 40459097 10.1056/NEJMoa2505133 PMC13292240

[72] Facon T , Dimopoulos MA , Leleu XP , Beksac M , Pour L , Hájek R , Liu Z , Minarik J , Moreau P , Romejko‐Jarosinska J *et al*. (2024) Isatuximab, Bortezomib, Lenalidomide, and dexamethasone for multiple myeloma. N Engl J Med 391, 1597–1609. 38832972 10.1056/NEJMoa2400712

[73] Einsele H *et al*. (2025) Modeling long‐term progression‐free survival in transplant‐eligible and transplant‐ineligible newly diagnosed multiple myeloma treated with daratumumab, bortezomib, lenalidomide, and dexamethasone. Oncol Res Treat 48, 13–364.

[74] Labanca C , Martino EA , Vigna E , Bruzzese A , Mendicino F , Lucia E , Olivito V , Puccio N , Neri A , Morabito F *et al*. (2025) Talquetamab in multiple myeloma: efficacy, safety, and future directions. Eur J Haematol 114, 386–399. 39604778 10.1111/ejh.14353 PMC11798766

[75] Lonial S , Popat R , Hulin C , Jagannath S , Oriol A , Richardson PG , Facon T , Weisel K , Larsen JT , Minnema MC *et al*. (2022) Iberdomide plus dexamethasone in heavily pretreated late‐line relapsed or refractory multiple myeloma (CC‐220‐MM‐001): a multicentre, multicohort, open‐label, phase 1/2 trial. Lancet Haematol 9, e822–e832. 36209764 10.1016/S2352-3026(22)00290-3

[76] Richardson PG , Badelita SN , Besemer B , Boudreault JS , Byun JM , Cerchione C , Gatt ME , Gibbs S , Koroda J , Martinez‐Lopez J *et al*. (2023) MM‐372 a phase III, two‐stage, randomized study of Mezigdomide, Bortezomib, and dexamethasone (MeziVd) versus Pomalidomide, Bortezomib, and dexamethasone (PVd) in relapsed/refractory multiple myeloma (RRMM): SUCCESSOR‐1. Clin Lymphoma Myeloma Leuk 23, S495–S496.

[77] Richardson PG , Amatangelo M , Berenson JR , Cerchione C , Dimopoulos MA , Toftmann Hansen C , Hwang SJ , Koo P , Kuroda J , Oriol A *et al*. (2023) A phase 3, two‐stage, randomized study of mezigdomide, carfilzomib, and dexamethasone (MeziKd) versus carfilzomib and dexamethasone (Kd) in relapsed/refractory multiple myeloma (RRMM): SUCCESSOR‐2. J Clin Oncol 41, TPS8070.

[78] Kleber M , Ntanasis‐Stathopoulos I & Terpos E (2023) The role of t(11;14) in tailoring treatment decisions in multiple myeloma. Cancers (Basel) 15, 5829. 38136374 10.3390/cancers15245829 PMC10742268

[79] Kumar S , Kaufman JL , Gasparetto C , Mikhael J , Vij R , Pegourie B , Benboubker L , Facon T , Amiot M , Moreau P *et al*. (2017) Efficacy of venetoclax as targeted therapy for relapsed/refractory t(11;14) multiple myeloma. Blood 130, 2401–2409. 29018077 10.1182/blood-2017-06-788786

[80] Bahlis NJ , Baz R , Harrison SJ , Quach H , Ho SJ , Vangsted AJ , Plesner T , Moreau P , Gibbs SD , Coppola S *et al*. (2021) Phase I study of Venetoclax plus Daratumumab and dexamethasone, with or without Bortezomib, in patients with relapsed or refractory multiple myeloma with and without t(11;14). J Clin Oncol 39, 3602–3612. 34388020 10.1200/JCO.21.00443 PMC8577687

[81] Bolarinwa A , Nagaraj M , Zanwar S , Abdallah N , Bergsagel PL , Binder M , Buadi F , Chhabra S , Cook J , Dingli D *et al*. (2025) Venetoclax‐based treatment combinations in relapsed/refractory multiple myeloma: practice patterns and impact of secondary cytogenetic abnormalities on outcomes. Blood Cancer J 15, 57. 40185701 10.1038/s41408-025-01264-2 PMC11971353

[82] Quach H , Popat R , Chen W , Kwok M , Yee A , Jiang Z , Xavier F , Callander N , Ho MY , Bal S *et al*. (2025) Initial phase 1b/2 study results with sonrotoclax (BGB‐11417) in combination with carfilzomib and dexamethasone in patients with t(11;14)‐positive relapsed/refractory multiple myeloma. Blood 146, 102.

[83] Petillo S , Capuano C , Molfetta R , Fionda C , Mekhloufi A , Pighi C , Antonangeli F , Zingoni A , Soriani A , Petrucci MT *et al*. (2021) Immunomodulatory effect of NEDD8‐activating enzyme inhibition in multiple myeloma: upregulation of NKG2D ligands and sensitization to natural killer cell recognition. Cell Death Dis 12, 836. 34482362 10.1038/s41419-021-04104-w PMC8418610

[84] Shah JJ , Jakubowiak AJ , O'Connor OA , Orlowski RZ , Harvey RD , Smith MR , Lebovic D , Diefenbach C , Kelly K , Hua Z *et al*. (2016) Phase I study of the novel investigational NEDD8‐activating enzyme inhibitor Pevonedistat (MLN4924) in patients with relapsed/refractory multiple myeloma or lymphoma. Clin Cancer Res 22, 34–43. 26561559 10.1158/1078-0432.CCR-15-1237 PMC5694347

[85] Bories P , Gasparetto C , Dingli D , Lonial S , Usmani SZ , Berdeja JG , Chung A , Afrough A , Rosiñol L , Yee AJ *et al*. (2024) First results from the dose escalation part of the phase 1 study of KTX1001, an Oral, first‐in‐class, potent inhibitor of MMSET/NSD2 for relapsed/refractory multiple myeloma (RRMM). Blood 144, 3370.

[86] Gunnell A , Kimber ST , Houlston R & Kaiser M (2025) NSD2‐epigenomic reprogramming and maintenance of plasma cell phenotype in t(4;14) myeloma. Oncotarget 16, 220–229. 40116454 10.18632/oncotarget.28706 PMC11927793

[87] Lauring J , Abukhdeir AM , Konishi H , Garay JP , Gustin JP , Wang Q , Arceci RJ , Matsui W & Park BH (2008) The multiple myeloma associated MMSET gene contributes to cellular adhesion, clonogenic growth, and tumorigenicity. Blood 111, 856–864. 17942756 10.1182/blood-2007-05-088674 PMC2200833

[88] Nandana D , Flynt E , Choo H , Hasheminasab A , Baker W , Gamble V , Kazeroun M , Sridharan V , Munshi N , Samur M *et al*. (2025) Anti‐adhesion properties of KTX‐1001, a selective NSD2/MMSET inhibitor, enhance Carfilzomib sensitivity in multiple myeloma. Clin Lymphoma Myeloma Leuk 25, S213–S214.

[89] Searle E , Cavet J , Campbell V , Knapper S , Bygrave C , el‐Sharkawi D , Pawlyn C , Creignou M , Walter H , Valcárcel D *et al*. (2023) Tolerability and clinical activity of novel first in class Oral agent, Inobrodib (CCS1477), in combination with Pomalidomide and dexamethasone in relapsed/refractory multiple myeloma. Blood 142, 2005.

[90] Nicosia L , Spencer GJ , Brooks N , Amaral FMR , Basma NJ , Chadwick JA , Revell B , Wingelhofer B , Maiques‐Diaz A , Sinclair O *et al*. (2023) Therapeutic targeting of EP300/CBP by bromodomain inhibition in hematologic malignancies. Cancer Cell 41, 2136–2153. 37995682 10.1016/j.ccell.2023.11.001

